# The Multifaceted Health Benefits of Broccoli—A Review of Glucosinolates, Phenolics and Antimicrobial Peptides

**DOI:** 10.3390/molecules30112262

**Published:** 2025-05-22

**Authors:** Celia María Curieses Andrés, José Manuel Pérez de la Lastra, Elena Bustamante Munguira, Celia Andrés Juan, Eduardo Pérez-Lebeña

**Affiliations:** 1 Hospital Clínico Universitario de Valladolid, Avenida de Ramón y Cajal, 3, 47003 Valladolid, Spain; cmcuriesesa@saludcastillayleon.es (C.M.C.A.); ebustamante@saludcastillayleon.es (E.B.M.); 2 Institute of Natural Products and Agrobiology, CSIC-Spanish Research Council, Avda. Astrofísico Francisco Sánchez, 3, 38206 San Cristóbal de La Laguna, Tenerife, Spain; 3 Cinquima Institute, Department of Organic Chemistry, Faculty of Sciences, Valladolid University, Paseo de Belén, 7, 47011 Valladolid, Spain; 4 Sistemas de Biotecnología y Recursos Naturales, 47625 Valladolid, Spain; info@glize.eu

**Keywords:** broccoli, bioactive compounds, sulforaphane, chemical synthesis, enantioseparation, sulforaphane diastereomer, HPLC

## Abstract

Broccoli, a highly valued Brassica vegetable, is renowned for its rich content of bioactive substances, including glucosinolates, phenolic compounds, vitamins, and essential minerals. Glucosinolates (GSLs), secondary plant metabolites, are particularly abundant in broccoli. The global consumption of broccoli has increased due to its high nutritional value. This review examines the essential bioactive compounds in broccoli and their biological properties. Numerous in vitro and in vivo studies have demonstrated that broccoli exhibits various biological activities, including antioxidant, anticancer, antimicrobial, anti-inflammatory, anti-obesity and antidiabetic effects. This review analyzes several aspects of the chemical and biological activity of GSLs and their hydrolysis products, isothiocyanates such as sulforaphane, as well as phenolic compounds. Particular emphasis is placed on sulforaphane’s chemical structure, the reactivity of its isothiocyanate fraction (-NCS), and given the different behavior of SFN enantiomers, a wide and detailed review of the chemical synthesis methods described, by microbial oxidation, or using a chiral ruthenium catalyst and more widely using chiral auxiliaries for synthesizing sulforaphane enantiomers. In addition, the methods of chiral resolution of racemates by HPLC are reviewed, explaining the different chiral fillers used for this resolution and a third section on resolution using the formation of diastereomeric complexes and subsequent separation on achiral columns. Additionally, this review highlights the presence of antimicrobial peptides in broccoli, which have shown potential applications in food preservation and as natural alternatives to synthetic antibiotics. The antimicrobial peptides (AMPs) derived from broccoli target bacterial membranes, enzymes, oxidative stress pathways and inflammatory mediators, contributing to their effectiveness against a wide range of pathogens and with potential therapeutic applications.

## 1. Introduction

**Figure 1:**
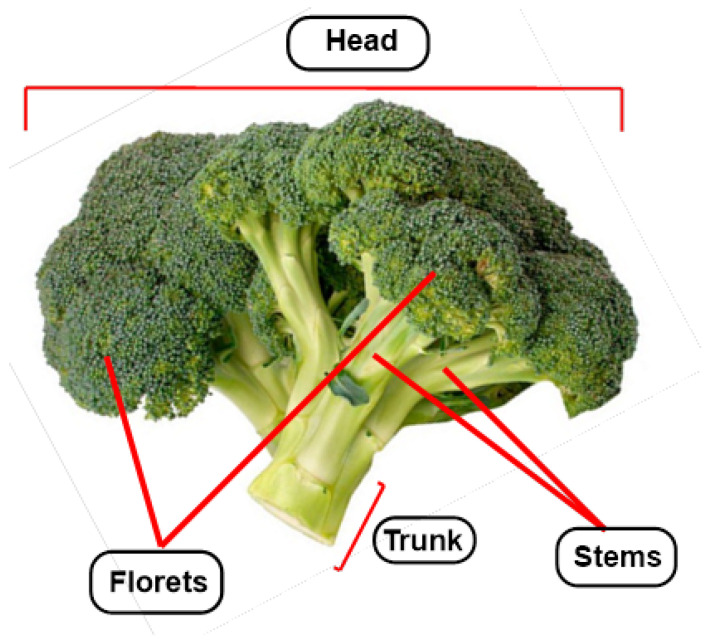
Broccoli (*Brassica oleracea* L. var. *italica*) anatomy.

*Brassica oleracea* L. var. *italica*, commonly known as broccoli, is an edible plant and represents one of the most important horticultural crops of the *Brassicaceae* family, to which other species of interest in agriculture such as cabbage, Brussels sprouts, cauliflower, radish, cabbage and arugula, among others, also belong. Broccoli can reach a height between 60 and 90 cm, 60 cm in diameter and a weight of 700 g [1]. The leaves make up the majority of the mature plant (47%), followed by the stems (21%), roots (17%) and inflorescences (15%) [2] (Figure 1).

The edible parts of broccoli are its immature flower heads (inflorescences) and the adjacent short, tender stems. This highly nutritious vegetable, originating from Western Asia and Europe, is widely consumed for its health benefits. Broccoli is an increasingly popular vegetable. According to FAO data, the greatest worldwide production of broccoli is centered in Asia, a continent that accounts for 79% of the production of this vegetable, with China being the main broccoli-producing nation in the world, producing an average of 9 million tons per year. This is followed by India as the second largest producer on the same continent, with an annual average of 8 million tons. The largest contributor to broccoli and cauliflower production in America is the United States, with a production of 1 million tons of this vegetable group in 2020. The same position in Europe is held by Spain, with an annual production in 2020 of 746,000 tons of these vegetables. Broccoli can be eaten fresh or prepared using various methods, both at home and industrially, including blanching, steaming, sautéing, baking and traditional boiling [3].

**Figure 2:**
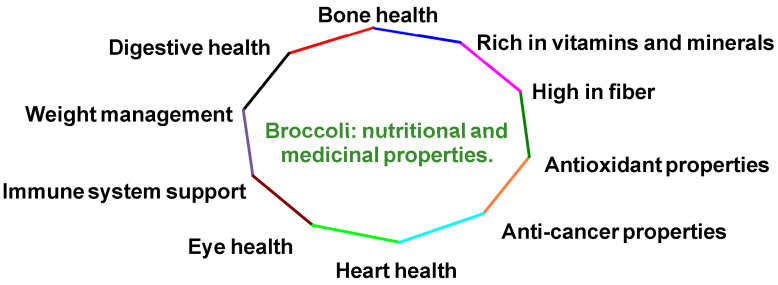
Summary of nutritional and health benefits of broccoli.

Many species in broccoli’s plant family are crucial components of human diets globally [4]. Regular consumption of these vegetables offers numerous health advantages and specifically provides several benefits (Figure 2):

- It contains abundant vitamins C, K and A, along with minerals such as potassium, calcium and iron. These nutrients act as antioxidants, helping to protect the body from oxidative stress and reducing inflammation [5,6].
- It is rich in dietary fiber, which supports digestion, increases feelings of fullness and contributes to a healthy digestive system [6].
- It provides various antioxidants, including vitamins C and E, β-carotene and several flavonoids, which help shield cells from free radical damage [7].
- It possesses potential anti-cancer properties due to its glucosinolate content, which the body can transform into cancer-fighting compounds [8,9,10].
- It supports heart health by potentially lowering cholesterol levels and helping maintain healthy blood pressure [9,11].
- It promotes eye health through its high vitamin A content and the presence of antioxidants [12].
- It boosts the immune system while supporting collagen production, wound healing and iron absorption [13].
- It contributes to bone health as it is a significant source of calcium and contains vitamin K [14].
- It aids in weight management due to its low-calorie content and high fiber levels [15].
- It supports digestive health, with its high fiber content promoting regular bowel movements [16].

Broccoli boasts an impressive nutritional profile and contains a significant number of bioactive compounds, also known as secondary metabolites, phytochemicals or phytonutrients. These bioactive compounds offer health benefits beyond basic nutrition. While not essential for human health, these phytochemicals can positively influence various disease processes and play a crucial role in long-term well-being [17].

The cultivation of broccoli is mainly focused on food consumption; the co-edible part comprises only 15% of the whole plant. This portion includes the florets, often accompanied by tender stems and sprouts. Broccoli production generates a substantial number of by-products, such as leaves and stems, which are generally discarded because they are considered unusable or commercially under-utilized; therefore, it is necessary to consider the revalorization of these by-products. These strategies can be focused on obtaining new ingredients for the food, cosmetics and pharmaceutical industries [18,19].

The primary bioactive compounds found in broccoli include phenols, glucosinolates, carotenoids, tocopherols, ascorbic acid and peptides. These compounds are renowned for their antioxidant properties and are associated with the health benefits of broccoli consumption. However, it is important to note that broccoli has a limited shelf life of only a few days. Without proper storage conditions, the phytochemical content can decrease rapidly [20].

The United Nations’ Food and Agriculture Organization (FAO) has recognized broccoli as one of the most nutritionally valuable vegetables. This classification is based on broccoli’s high content of health-promoting compounds (such as glucosinolates and phenolic compounds) and its significant contribution of vitamins, minerals and fiber to the diet (FAO, 2021) [21].

## 2. Bioactive Compounds in Broccoli

The health benefits of broccoli extend beyond its primary nutrients to include secondary plant metabolites known as bioactive phytochemicals. These compounds include phenolic compounds (which possess antioxidant properties and help neutralize free radicals, such as flavonoids, hydroxycinnamic acids and their derivatives), carotenoids (lutein, zeaxanthin, β-carotene, violaxanthin and neoxanthin, which act as powerful antioxidants and support eye health), chlorophylls (which contribute to the green color of broccoli and have potential health-promoting effects), glucosinolates (sulfur-containing compounds that can be converted into cancer-fighting substances in the body, such as sulforaphane (SFN)), vitamins (particularly vitamins A, C and K, which contribute to various health benefits), essential minerals (including selenium, potassium and manganese, which play crucial roles in various bodily functions) and antimicrobial peptides (which have shown potential applications in food preservation and as natural alternatives to synthetic antibiotics) [22].

These bioactive phytochemicals work synergistically to provide various health benefits, including antioxidant, anti-inflammatory, anticancer and antimicrobial activities. The concentration and efficacy of these compounds in broccoli can vary depending on factors such as genotype, growing conditions, harvest time and plant part (e.g., florets, leaves, or stems) [23].

### 2.1. Glucosinolates

Glucosinolates, also known as thioglycosides, are nitrogen–sulfur-containing secondary metabolites found almost exclusively in plants of the Brassicaceae family, with broccoli being one of the richest sources. These bioactive compounds are considered the hallmark of broccoli due to their high concentration. Glucosinolates are particularly abundant in broccoli seeds and sprouts during their early developmental stages, where their levels can be up to ten times higher than those found in mature broccoli. These compounds are responsible for the distinctive pungent aroma and bitter taste of Brassicaceae vegetables and serve as natural defense mechanisms for the plants [24].

The chemical structure of glucosinolates (β-D-thioglycoside-N-hydroxysulfate) consists of three main components: (i) a β-D-thioglucose group, (ii) a sulfonated oxime group with a C=N double bond in the Z configuration, confirmed through X-ray crystallography and (iii) a R side chain derived from amino acids such as methionine, phenylalanine, tryptophan, or branched-chain amino acids [25,26].

**Figure 3:**
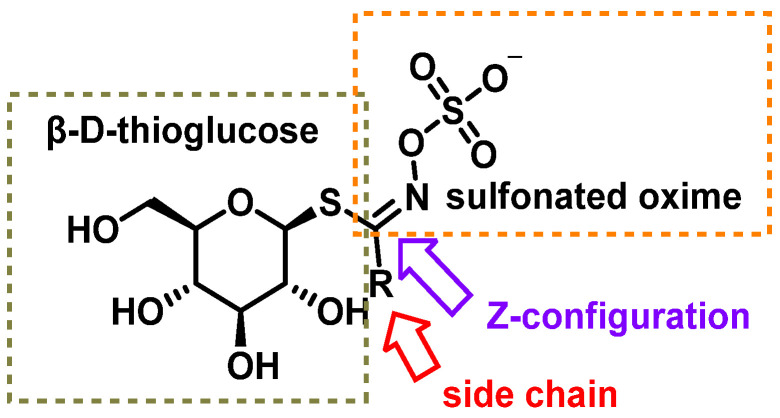
Glucosinolate structure.

When glucosinolates are hydrolyzed by the enzyme myrosinase, glucose and thiohydroxymate-O-sulfate are released. This reaction is followed by an intermolecular Lossen rearrangement, resulting in the formation of isothiocyanate (RN=C=S), a compound known for its potential health-promoting properties, including anticancer effects [27] (Figure 3).

Glucosinolates (GSLs) are chemically stable molecules under standard conditions, with their breakdown primarily occurring through enzymatic action. These compounds coexist with their hydrolyzing enzyme, myrosinase (a β-thioglucosidase), within plant cells but remain segregated in distinct cellular compartments. GSLs remain inert until contact with myrosinase occurs via physical disruption, such as chewing, food processing, or pest damage [28].

Humans lack endogenous myrosinase to convert GSLs into bioactive isothiocyanates (ITCs); however, the gut microbiota—particularly certain *Bifidobacterium* species—can perform this conversion 2–3 h after consuming *Brassica* vegetables. Hydrolysis occurs via plant-derived myrosinase, which activates GSLs in the small intestine, or bacterial myrosinase, which facilitates hydrolysis in the colon [29].

GSLs only become biologically active when hydrolyzed to ITCs. These metabolites are absorbed in the small intestine and colon, with detectable levels appearing in human urine within 2–3 h of Brassica consumption.

**Figure 4:**
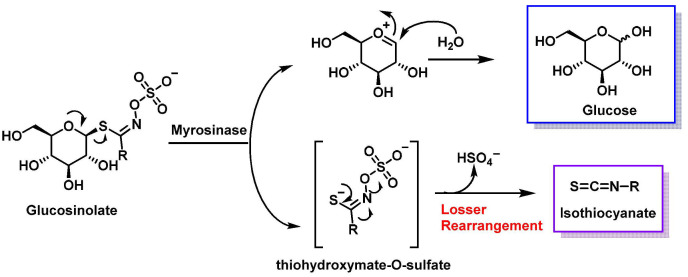
Hydrolysis of glucosinolates by myrosinase (an enzyme found in plants and intestinal microflora) to form isothiocyanates and glucose.

In the hydrolysis mechanism (Figure 4), myrosinase cleaves the thioglycosidic bond, releasing β-D-glucose and forming an unstable thiohydroxymate-O-sulfonate intermediate. This compound undergoes pH-dependent transformations: at neutral conditions (pH~7), the Lossen rearrangement produces ITCs (R-NCS), and at acidic conditions (pH~4), decomposition yields nitriles instead of ITCs. At low pH, the Lossen rearrangement is inhibited, favoring nitrile formation over ITCs [30].

Thiohydroxymate-O-sulfate—the unstable organic aglycone derived from glucosinolate hydrolysis—undergoes structural rearrangements influenced by parent glucosinolate structure, hydrolysis conditions (pH, temperature), cofactor presence (e.g., Fe^2+^ ions) and regulatory proteins (e.g., epithiospecifier protein (ESP), thiocyanate-forming protein (TFP)). These factors determine the final degradation products, which fall into isothiocyanates (ITCs), thiocyanates, nitriles, epithionitriles (EPT) and oxazolidine-2-thiones [31].

**Figure 5:**
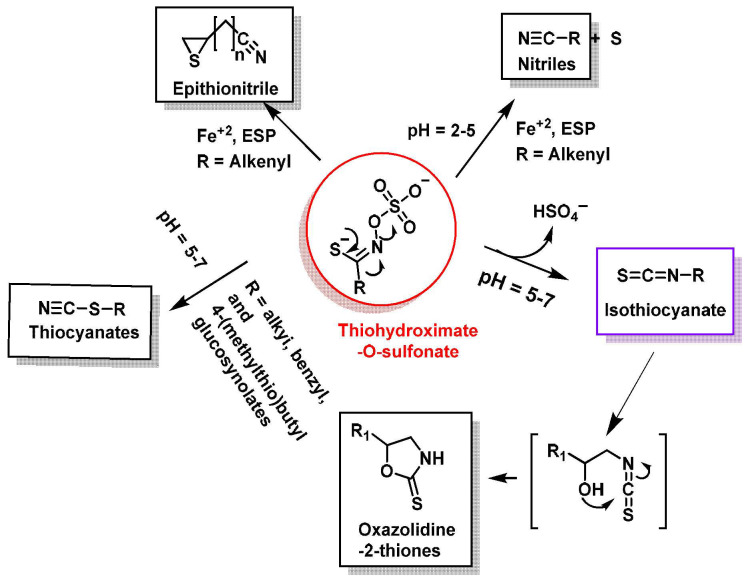
Chemical structure of the different thiohydroxymate-O-sulphate decomposition compounds.

ESP-mediated pathways direct the conversion of alkenyl glucosinolates (GLs) to epithionitriles and non-alkenyl GLs to nitriles. This protein acts as a myrosinase cofactor, steering reactions toward specific byproducts (Figure 5). In ESP’s presence, intermediates like thiocyanate (RSC≡N) or oxazolidin-thione transform into epithionitriles (alkenyl GLs) or nitriles (non-alkenyl GLs) [31,32,33].

Over 130 distinct glucosinolates (GSLs) have been identified and characterized. These compounds are typically classified based on the chemical structure of their R side chains [34,35]. For instance, GSLs isolated from broccoli varieties are grouped into three categories according to their precursor amino acids:

(i) Aliphatic GSLs, derived from methionine, isoleucine, leucine, or valine [36,37,38,39,40,41,42,43,44,45,46].
(ii) Aromatic GSLs, originating from phenylalanine or tyrosine [47,48,49].
(iii) Indole GSLs, synthesized from tryptophan [50,51].

**Figure 6:**
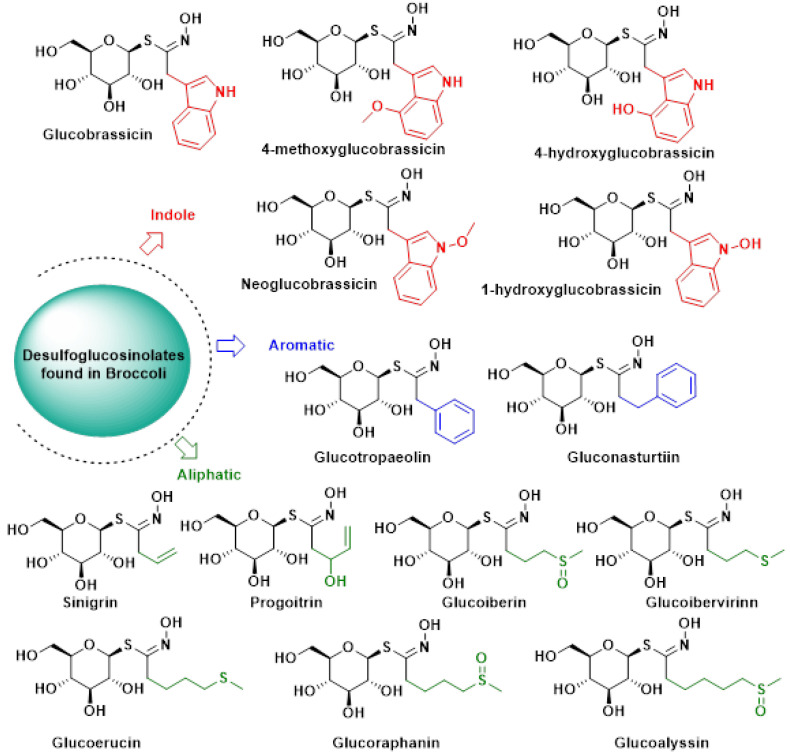
Structures of desulfoglucosinolates found in Broccoli.

A more recent classification system, proposed by Blaževic et al., categorizes GSLs based on the presence or absence of aromatic motifs in their molecular structure [35] (Figure 6).

Of the more than 100 identified glucosinolates (GSLs), approximately 50 are commonly found in Brassica genus vegetables. Typically, each plant contains 3 to 4 predominant GSLs, though up to 15 different types have been observed in some species [52]. The diversity in GSL side chains leads to a wide array of sulfur-containing derivatives through enzymatic breakdown [53].

In broccoli, the prevalent glucosinolates include glucoraphanin (4-methylsulfinylbutyl), sinigrin, progoitrin (2-hydroxy-3-butenyl), gluconapine (3-butenyl), glucobrassicin, neoglucobrassicin (1-methoxy-3-indolylmethyl) and glucoraphanin (the most abundant, comprising over 50% of total glucosinolates, while sinigrin is present in comparatively lower amounts) [54].

**Figure 7:**
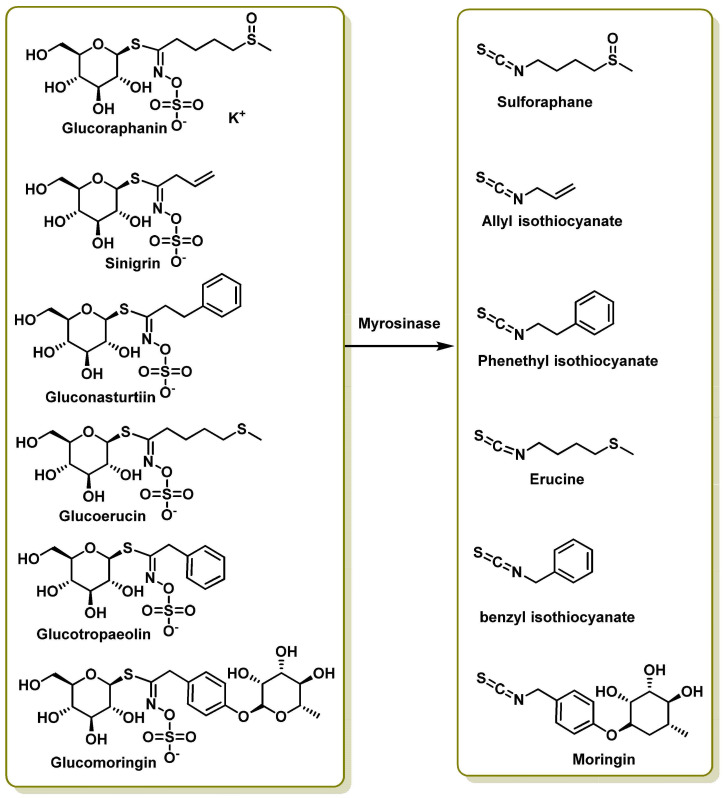
Examples of isothiocyanates and their glucosinolate precursors.

Key glucosinolates and their corresponding hydrolysis products include glucoraphanin (GRA), sulforaphane (SFN), sinigrin (SIN), allyl isothiocyanate (AITC), gluconasturtiin (GST), phenethyl isothiocyanate (PEITC), glucoerucin (GER), erucin (ER), glucotropaeolin (GTL), benzyl isothiocyanate (BITC), glucomoringin (GMG) and moringin (MG). Figure 7 illustrates these transformations [55,56].

#### 2.1.1. Bioavailability of Glucosinolates

In cruciferous vegetables, the composition and content of glucosinolates are relatively stable, but depend on the genus and species, and may vary with plant growth, with different parts of a plant (e.g., in seeds, roots or leaves), post-harvest storage conditions and culinary processing [52,57]. Taking these variables into account, the amounts of isothiocyanates formed from glucosinolates in foods are variable.

Most cruciferous vegetables are cooked before consumption; the bacterial myrosinase in the intestine, rather than vegetable myrosinase, is responsible for the initial process of glucosinolate degradation. The intensity of heat denaturation is important when the temperature applied is high and the cooking time is long [58]. Inactivation may occur due to cooking at 60 °C [59], sautéing [60], microwaving [61] or other forms of heating or processing of cruciferous vegetables [58]. When myrosinase is inactivated, glucosinolates transit to the colon, due to their hydrophilic nature (thioglucose and sulphate group), and are metabolized by the intestinal microbiota. For example, intact glucosinolates may be partially absorbed in the stomach, and the remaining glucosinolates will transit through the gastrointestinal tract to reach the small intestine where they may be hydrolyzed by plant myrosinase and the degradation products may be absorbed. The remaining unhydrolyzed glucosinolates will then transit to reach the colon where they may be hydrolyzed by bacterial myrosinase, and the degradation molecules generated will be absorbed and/or excreted.

Johnson et al., 2002, reported that isothiocyanates were absorbed through the small intestine and colon, where their metabolites were detected 2–3 h after consumption [62].

Detailed studies of the effect of cooking Brassica vegetables on the absorption of isothiocyanates have been carried out [52]. Vermeulen et al., 2008, found that the consumption of raw broccoli resulted in faster absorption, higher bioavailability and higher maximum plasma amounts of sulforaphane compared to cooked broccoli [63]. Higher amounts of sulforaphane were found in blood and urine when broccoli was eaten raw (bioavailability 37%) compared to cooked (3.4%, *p* = 0.002). Sulforaphane absorption was delayed when cooked broccoli was consumed (peak plasma time = 6 h) compared to raw broccoli (1.6 h, *p* = 0.001). Excretion half-lives were comparable, 2.6 and 2.4 h on average, for raw and cooked broccoli, respectively (*p* = 0.5) [63].

Conaway et al., 2000, observed that isothiocyanate levels were three times higher in urine after consumption of raw broccoli compared to steamed broccoli (32.3% vs. 10.2%). These authors compared the metabolic fate of glucosinolates after the ingestion of fresh and steamed broccoli [64].

Fahey et al., 2015, also demonstrated that active myrosinase was critical for the bioavailability of glucoraphanin in broccoli extracts as sulforaphane was three to four times more bioavailable in extracts with an active myrosinase enzyme than in extracts without [65].

Ghawi et al., 2013, found that the addition of mustard seeds, which contain a myrosinase isoform that is much more heat stable than other brassicas, to thermally processed broccoli increased sulforaphane formation by three to five times [66].

#### 2.1.2. Biosynthesis of Glucosinolates in Plants

Glucosinolate (GSL) biosynthesis originates from α-amino acid metabolism and can be divided into three phases [34,67].

(1) Elongation of the side chain of amino acids.

The initial reaction occurs in the cytosol and the subsequent elongation steps take place in chloroplasts. The process unfolds as follows:

- Deamination of aliphatic or aromatic amino acids, yielding 2-oxo acids;
- Condensation of 2-oxo acids with Acetyl-CoA, forming 2-malate derivatives;
- Isomerization of 2-malate to 3-malate;
- Oxidation and decarboxylation, resulting in the loss of the initial amino acid carboxyl group and the formation of an elongated 2-oxo acid molecule.

**Figure 8:**
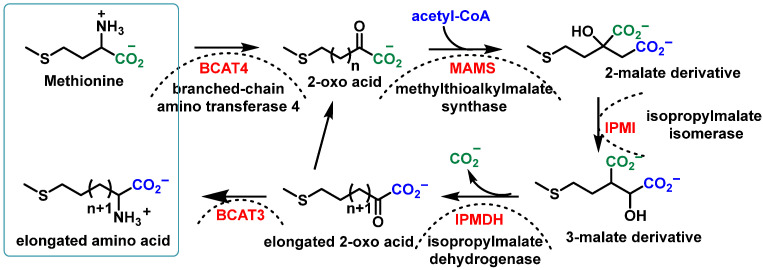
Aliphatic glucosinolate chain elongation.

The elongated 2-oxo acid can then re-enter the elongation cycle or undergo transamination to form an elongated amino acid. Figure 8 illustrates this process [49].

(2) Synthesis of GSL from the modified amino acid

The biosynthesis of glucosinolates (GSLs) involves a multi-stage enzymatic pathway that transforms amino acids into diverse sulfur-containing secondary metabolites. This process includes chain elongation, core structure assembly, and secondary modifications, with specific enzymes catalyzing each step. The key stages are as follows: (i) conversion of amino acids to aldoximes, cytochrome P450 monooxygenases oxidize amino acids (e.g., methionine, phenylalanine) into aldoximes (CYP79 family, catalyzing the oxidation of parent amino acids or their chain-elongated derivatives); (ii) formation of reactive intermediates, due to CYP83 family enzymes further oxidize aldoximes to generate reactive aci-nitro or nitrile oxide compounds, critical for sulfur conjugation in the next step; (iii) sulfur conjugation with glutathione (GSH), where GSH, not cysteine, donates sulfur for conjugation, in a process in which activated aldoximes bind to GSH via enzymatic action, forming S-alkylthiohydroxymate conjugates; (iv) cleavage (the C-S lyase enzyme SUR1 cleaves the GSH conjugate to produce thiohydroxymates) and glycosylation (UDP-glucose:thiohydroxymic acid S-glucosyltransferase (UGT74B1) adds a glucose moiety to form desulfoglucosinolates); and (v) final sulfation, where desulfoglucosinolate sulfotransferases catalyze sulfation, converting desulfoglucosinolates into bioactive GSLs [24,68,69,70].

**Figure 9:**
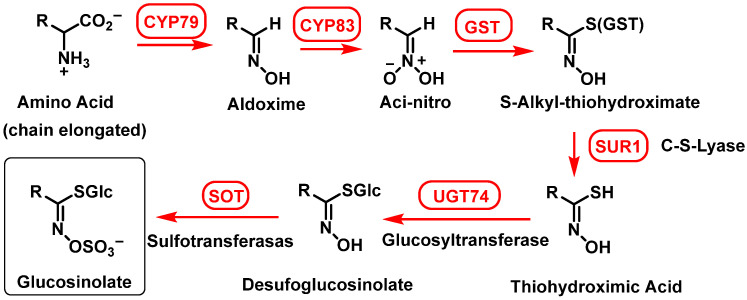
Glucosinolate core biosynthesis.

Transferases involved in forming the GSL core structure have been identified and extensively documented in the scientific literature [71,72] (Figure 9).

(3) GSL sidechain modifications

The third stage of glucosinolate (GSL) biosynthesis is crucial for increasing the diversity of GSLs found in nature. After the formation of parent glucosinolates, a wide range of side chain modifications occur, including oxidation, esterification, hydroxylation, methoxylation, alkenylation and benzoylation. These modifications give rise to the various GSLs observed in plants. The biological activity of GSLs is largely determined by their side chain structure, making these secondary modifications particularly interesting for potential applications [73].

For aliphatic glucosinolates, secondary modifications include oxygenations, hydroxylations, alkenylations, and benzoylations. Indole glucosinolates commonly undergo hydroxylations and methoxylations. A common modification, the S-oxygenation of aliphatic glucosinolates, is carried out by flavin monooxygenases (FMO GS-OXs). Alkenyl glucosinolates, such as sinigrin, are produced from S-oxygenated glucosinolates by 2-oxoglutarate-dependent dioxygenases (AOPs).

In *Arabidopsis thaliana*, the GSL-ALK locus controls the conversion of methylsulfinyl glucosinolates to alkenyl glucosinolates, which is related to the production of sinigrin and gluconapin. The BoGSL-ALK gene, which has been cloned, has been shown to influence side-chain modifications in glucosinolate biosynthesis. These secondary modifications play a key role in determining the final structure and biological activity of glucosinolates, contributing to their diverse functions in plant defense and human nutrition [74].

## 3. Most Studied Broccoli Glucosinolate Hydrolysis Compounds

The hydrolysis of glucosinolates (GSLs) in broccoli primarily produces isothiocyanates (ITCs), which are more bioactive than their GSL precursors. Among these, glucoraphanin (GR), a precursor to the isothiocyanate SFN, is the most extensively studied glucosinolate in broccoli. Indole glucosinolates, on the other hand, yield unstable thioketone intermediates (TKIs), which predominantly break down into indole-3-carbinol (I3C) and 3,3′-diindolylmethane [75,76].

### 3.1. Sulforaphane

SFN, chemically known as 1-isothiocyan-4-(methylsulfinyl) butane, is a low-toxicity phytochemical derived from its inactive precursor glucoraphanin, which constitutes 35–50% of the total glucosinolates in broccoli. SFN has garnered significant attention for its potential use in clinical applications and as a dietary supplement [77].

The conversion of glucoraphanin to SFN is catalyzed by myrosinase, an enzyme present in broccoli tissues. Broccoli sprouts are particularly rich in SFN content. Research has demonstrated that SFN acts as a potent natural antioxidant and free radical scavenger. It also activates the nuclear factor erythroid 2-related factor 2 (Nrf2) signaling pathway, which plays a critical role in cellular defense mechanisms against oxidative stress [78].

Key mechanisms of SFN action involve the activation of MAPKs (SFN activates three mitogen-activated protein kinases (MAPKs): extracellular signal-regulated protein kinase (ERK), c-Jun N-terminal kinase (JNK), and p38 MAPK), activation of PKC (SFN stimulates protein kinase C (PKC), which directly phosphorylates Nrf2) and PI3K/AKT pathway, because SFN activates phosphatidylinositol 3-kinase (PI3K) and protein kinase B (AKT), leading to Nrf2 activation [79].

Upon activation, Nrf2 undergoes phosphorylation, translocates to the nucleus, and binds to the antioxidant response element (ARE). This interaction induces the transcription of cytoprotective genes that reduce oxidative stress. Exposure to ARE inducers inhibits Nrf2 ubiquitination, allowing it to accumulate in the nucleus. Here, Nrf2 dimerizes with small Maf proteins and binds to ARE sequences, stimulating the expression of genes encoding enzymes such as heme oxygenase 1 (HO-1), quinone oxidoreductase 1 (NQO1), and glutathione S-transferases (GST) [79,80].

**Figure 10:**
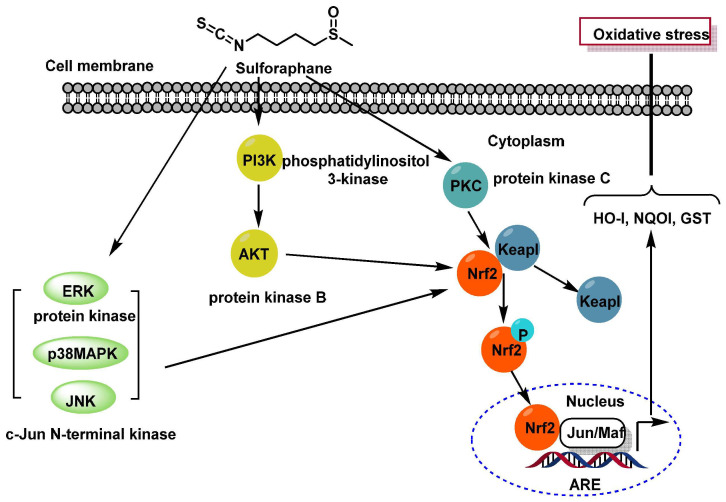
SFN activation of Nrf2 signaling.

Keap1, a regulatory protein that normally sequesters Nrf2 in the cytoplasm, releases it under oxidative stress conditions, enabling Nrf2 to bind to AREs and initiate gene transcription. This cascade significantly enhances cellular protection against oxidative damage [81,82] (Figure 10).

SFN exhibits a wide range of beneficial effects beyond its anti-inflammatory and antimicrobial properties [83]. These include anticancer activity, antidiabetic effects [84,85], anti-obesogenic and anti-obesity properties, and cardioprotective effects [81,86,87,88]. The SFN content in broccoli has been quantified as follows: broccoli seeds contain 396.18 mg/100 g fresh weight, while broccoli sprouts contain 391.81 mg/100 g dry weight [19,89].

SFN’s anticancer mechanisms operate simultaneously on multiple cellular targets. It protects DNA by inhibiting mutagenic factors (phase I) and activating phase II detoxification enzymes. Additionally, SFN inhibits cancer cell proliferation and activates apoptosis, limiting the multiplication of mutated cancer cells and inhibiting neogenesis and metastasis. SFN has demonstrated the ability to prevent, remove, and reverse preneoplastic lesions. Researchers have investigated the primary anticancer mechanisms of SFN and determined the concentrations required for their activation, as outlined in Table 1 [90].

SFN’s anticancer activity is multifaceted, involving the inhibition of cancer cell proliferation, arrest of the cell cycle, and enhancement of apoptosis. It alters several epigenetic and non-epigenetic mechanisms, including the inhibition of histone deacetylase (HDAC) activity, halting histone phosphorylation, downregulation of DNA methyltransferases (DNMTs), and regulation of noncoding RNAs [91].

Beyond its anticancer effects, SFN has shown promise in addressing other health concerns. For instance, it has antidiabetic effects, improving blood sugar levels by suppressing hepatic glucose production and enhancing glucose tolerance. SFN also exhibits anti-obesogenic and anti-obesity properties by improving glucose tolerance and reducing fat accumulation, partly through its ability to activate Nrf2, a transcription factor regulating oxidative stress and inflammation in cells [92].

Furthermore, SFN has demonstrated cardioprotective properties. It improves the viability of cardiomyocytes, diminishes apoptotic cells, suppresses caspase-3 activity, alleviates damage to mitochondrial membrane potential, and decreases the expression of ER stress-related apoptosis proteins. Additionally, SFN inhibits foam cell formation and atherosclerosis by regulating macrophage cholesterol transport and accumulation [93].

These diverse effects highlight SFN’s potential as a therapeutic agent for various health conditions, including cancer, diabetes, obesity and cardiovascular disease.

Fimognari et al., 2007, studied the main anticancer mechanisms of SFN and the concentrations required for their activation (Table 1) [90].

According to the U.S. National Cancer Institute, SFN is one of the 40 most promising anticancer compounds. There are several excellent reviews on the anticancer activities as well as the mechanisms of action of SFN [94,95,96,97]. In the review conducted by Lukasz Janczewski, different methods of SFN synthesis are described [98].

#### 3.1.1. Sulforaphane Reactivity

**Figure 11:**
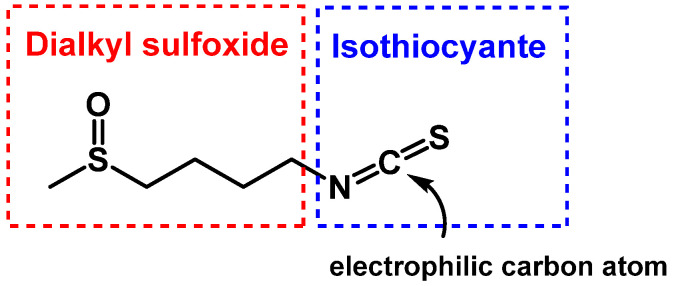
Chemical structure of the functional groups of SFN.

SFN has two reactive functional groups, a dikazkyl-sulfoxide and an isothiocyanate (Figure 11).

**Figure 12:**
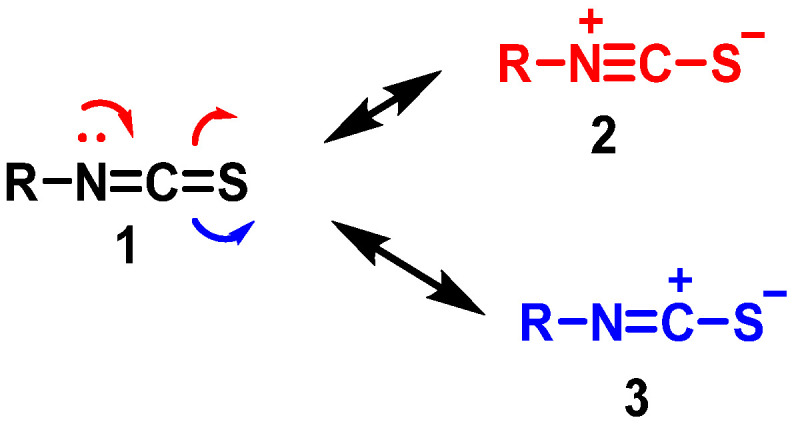
Mesomeric structures proposed for –NCS group.

The group –N=C=S has a linear structure that was proposed as early as 1929 by Perschke [99]. The isothiocyanate group presents the canonical structures 1–3 shown in the figure containing accumulated double bonds (1) and ionic structures with a triple (2) or double bond N-C (3) and a negative charge located in the sulfur atom (Figure 12).

SFN structure is influenced by the dipole moments of its canonical forms, specifically, structures 1 and 3 contribute most significantly to the -NCS group, while structure 2’s contribution is negligible. This structural aspect is important for understanding the reactivity and stability of SFN.

SFN is highly reactive and unstable due to its functional groups. Its production and stability are affected by several factors, including temperature, pH, and enzyme activity. These factors limit SFN’s practical applications, which is why there are no marketed SFN drugs. Instead, the SFN-related market primarily consists of broccoli extracts, anti-aging creams, dietary concentrates, and other herbal products.

**Figure 13:**
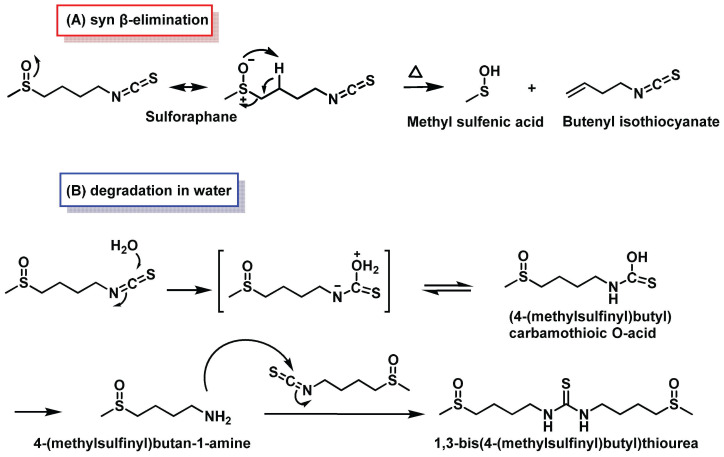
(**A**) β-elimination without, transforming SFN into a butenyl isothiocyanate and methylsulfenic acid. (**B**) Reaction with SFN and water.

At high temperatures, SFN’s sulfoxide group can undergo β-elimination, transforming SFN into butenyl isothiocyanate and methylsulfenic acid (Figure 13A) [100]. The isothiocyanate group in SFN is highly reactive towards nucleophiles due to its electrophilic central carbon. It readily reacts with sulfur, nitrogen, and oxygen nucleophiles. Common reactions include the formation of thiocarbamates with cysteine residues in proteins and glutathione (GSH), as well as alkylation reactions with α-amino groups in N-terminal protein residues, ε-amino groups of lysine, and secondary amines like proline. These reactions form thiourea products.

In aqueous and biological media, isothiocyanates react to form inactive, non-volatile organic compounds. Under physiological conditions, they react reversibly with thiols and irreversibly with amines. Notably, isothiocyanates react 1000 times faster with thiols than with amines. Their hydrolysis produces amines (Figure 13B) [101]. This reactivity profile highlights the complex interactions of SFN with biological molecules, which is crucial for understanding its potential health benefits and limitations [102].

#### 3.1.2. Interaction of Sulforaphane with Keap1

SFN demonstrates a high affinity for cysteine residues in proteins, forming thionoacyl adducts through its electrophilic properties. This interaction is particularly notable with Cys-151 in human Keap1, a key protein in the Nrf2 signaling pathway. However, SFN’s reactivity extends beyond this single residue, also modifying other sensing cysteines such as Cys-38, Cys-368, and Cys-489 in Keap1 [103,104,105].

**Figure 14:**
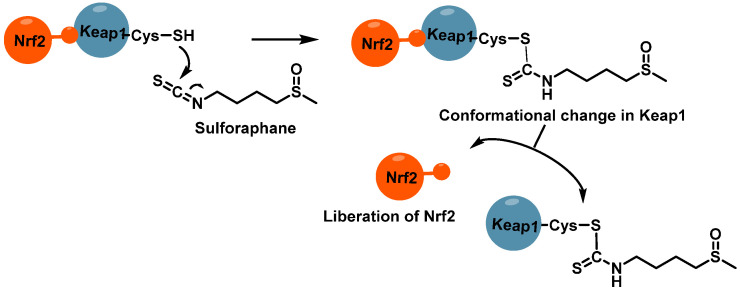
Interaction of SFN with Keap1.

The isothiocyanate group of SFN and its analogues exhibits rapid but reversible reactions with the sulfhydryl groups of Keap1 cysteines. This chemical interaction has significant implications for the Nrf2/Keap1 complex. By modifying these critical cysteine residues, SFN disrupts the normal functioning of the complex, effectively protecting Nrf2 from ubiquitination. This mechanism is illustrated in Figure 14, highlighting how SFN’s interaction with Keap1 leads to Nrf2 activation and subsequent cellular protective responses [106,107].

Interestingly, SFN’s ability to modify cysteine residues extends beyond its effects on the Nrf2/Keap1 pathway. Research has shown that SFN can also interact with cysteine residues in other proteins, such as those in the AP-1 transcription factor. Specifically, SFN has been found to inhibit AP-1 activity by interacting with Cys154 in cFos and Cys272 in cJun, which are part of the DNA-binding domain of these proteins [108].

This interaction contributes to SFN’s chemopreventive properties, particularly in reducing UVB-induced squamous cell carcinomas in animal models [109].

The versatility of SFN’s interactions with various cysteine-containing proteins underscores its potential as a multifaceted compound in cellular regulation and disease prevention. These interactions not only affect transcription factor activity but also influence various cellular processes, including apoptosis, cell migration, and invasion [110]. As research continues, the full scope of SFN’s cysteine-mediated effects on cellular function and its therapeutic potential are likely to be further elucidated.

#### 3.1.3. Interaction of Sulforaphane with GSH

SFN undergoes a complex metabolic transformation in the body through the mercapturic acid pathway, a process that is crucial for its eventual elimination and potential therapeutic effects. This pathway begins immediately after SFN ingestion and involves several enzymatic steps. The initial step in this metabolic process is the conjugation of SFN with glutathione (GSH). This reaction is catalyzed by glutathione transferase (GST), an enzyme that plays a key role in cellular detoxification processes. The electrophilic isothiocyanate group of SFN reacts with the nucleophilic -SH group of the cysteine residue in GSH, forming a dithiocarbamate. This reaction marks the beginning of the isothiocyanate metabolism, which is commonly referred to as the mercapturic acid pathway [111].

Following this initial conjugation, the SFN-GSH complex undergoes a series of successive cleavage reactions. These reactions are catalyzed by specific enzymes: γ-glutamyltranspeptidase, cysteinylglycinase, and N-acetyltransferase. The final product of this enzymatic cascade is SFN-N-acetylcysteine, also known as a mercapturic acid.

**Figure 15:**
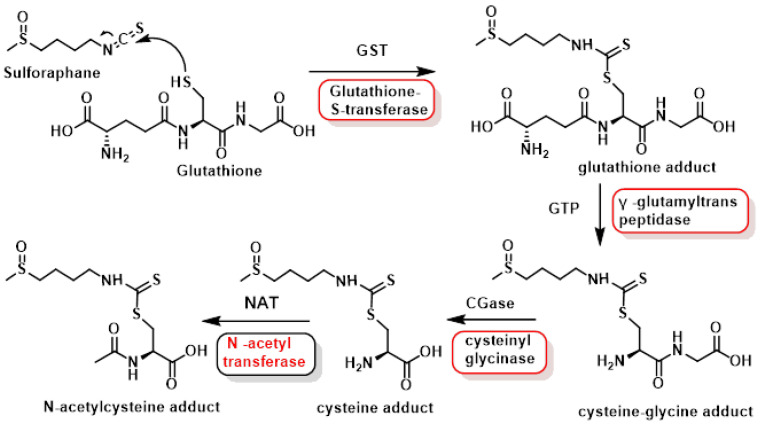
SFN is metabolized via the mercapturic acid pathway upon conjugation with glutathione and undergoes further biotransformation to produce different metabolites.

The formation of this mercapturic acid primarily occurs in the liver, which is the main site of drug metabolism in the body. Once formed, the SFN-N-acetylcysteine is transported to the kidneys. From there, it is eliminated from the body through urinary excretion. This entire process, from ingestion to excretion, is illustrated in Figure 15, providing a visual representation of SFN’s metabolic journey through the body [112].

Understanding this metabolic pathway is crucial for appreciating how SFN exerts its biological effects and how it is processed and eliminated by the body. This knowledge can inform future research into SFN’s potential therapeutic applications and help in developing strategies to enhance its bioavailability and efficacy [113].

Most SFN undergoes metabolic transformation through the mercapturic acid pathway before excretion. The main urinary metabolite is sulforaphane-N-acetylcysteine (SF-NAC). The excretion rate and percentage of SFN eliminated through urine vary depending on the form of administration. When consumed as fresh broccoli sprouts, about 60–65% of the ingested dose is excreted as SF-NAC within 24 h. For SFN-rich formulations, approximately 70% of the administered SFN is eliminated in 24 h. Overall, urinary excretion of SFN metabolites can range from 70% to 90% of the ingested dose. The elimination of SFN and its metabolites is indeed rapid, with most excretion occurring within the first 12–24 h after administration. Urinary concentrations of SFN equivalents are typically 2–4 orders of magnitude higher than those in plasma. This rapid metabolism and excretion pose challenges for maintaining therapeutic levels of SFN in the body [114,115].

Given these pharmacokinetic characteristics, developing appropriate drug delivery systems to increase SFN’s bioavailability and concentration at target sites is a valid consideration. Some approaches being explored include nanoencapsulation using broccoli membrane vesicles, which has shown improved cellular uptake and metabolism of SFN in cancer cells. Another promising approach involves gold-coated iron oxide nanoparticles functionalized with folic acid for targeted delivery to cancer cells [116].

These delivery systems aim to enhance SFN’s stability, absorption and therapeutic efficacy, potentially addressing the limitations posed by its rapid metabolism and excretion. By improving the pharmacokinetic profile of SFN, these novel delivery methods could enhance its potential as a therapeutic agent in various health conditions [117].

### 3.2. The Risks of Excessive Intake of Sulforaphane

**Figure 16:**
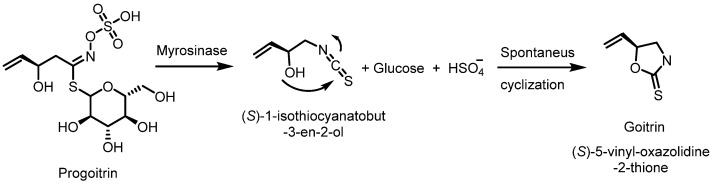
Progoitrin is biologically inactive, but upon hydrolysis by the enzyme myrosinase it is transformed into goitrin.

Sulforaphane is consumed as a component of cruciferous vegetables, and also as a dietary supplement or drug. Possible side effects, especially when consumed in large amounts or by supplementation, are summarized below:

- Gastrointestinal discomfort: Some people report digestive symptoms such as diarrhea, gas or stomach upset after consuming sulforaphane supplements or large amounts of broccoli and other cruciferous vegetables. These symptoms may be more pronounced if the digestive system is not accustomed to the fiber and compounds present in these vegetables. Therefore, sufferers of gastrointestinal disorders, such as irritable bowel syndrome, may experience worsening symptoms due to the fermentation of fiber in the intestinal tract [118].
- Allergic reactions. Although rare, some people may experience allergic reactions to sulforaphane or its food sources. In rare cases, symptoms can range from mild skin rashes to more severe reactions [119].
- Thyroid interaction: There are indications that sulforaphane may interfere with thyroid function, especially in people with hypothyroidism. This is due to the goitrogenic compounds present in cruciferous vegetables, which may affect iodine absorption and thus thyroid hormone production. These vegetables also contain 2-hydroxy-3-butenyl glucosinolate (progoitrin) and indole glucosinolate, which can be converted to goitrin and thiocyanate, which act as biocogens in animal models. The conversion can occur either by spontaneous cyclization, as in Figure 16, or activated by the enzyme myrosinase [120,121].

These dioxins can block the transport of iodine into the thyroid gland and inhibit the incorporation of iodine into thyroglobulin, which impairs the synthesis of active thyroid hormone. Some methods of food preparation, such as washing, soaking and boiling, help to reduce bocytogens in food by destroying myrosinase and preventing the release of bocytrin from progoitrin [120,121,122].

- Potential drug interactions. Sulforaphane potentiates the anticonvulsant efficacy of carbamazepine in a seizure test, indicating possible pharmacokinetic interactions. Sulforaphane may interact with drugs that are metabolized in the liver, particularly those that are substrates of cytochrome P450 enzymes, such as CYP3A4 and CYP1A2. The interaction between SFN and fu-rosemide, verapamil and ketoprofen modifies the activity of the enzyme system involved in drug metabolism and transport. This can lead to altered drug effectiveness and also to the development of multidrug resistance [123].
- Variable effects on tumorigenesis. Several studies with higher doses of sulforaphane in mice describe toxicities that require careful attention to risk-benefit analyses and determination of therapeutic or prophylactic indices. Shorey et al., 2013, observed increased morbidity and no reduction in lung tumorigenesis in offspring born to mothers receiving transplacental and lactational exposure to the carcinogen dibenzo[def,p]chrysene and supplemented with dietary sulforaphane (400 ppm) in contrast to many reports of chemoprotection in adult animal models [124].
- Tao et al., 2018, used a vinyl carbamate chemical carcinogenesis model (A/J mice) and a genetic model (LSL-K-rasG12D/+ mice) to induce lung cancers [125]. In the genetic model, pretreatment with SF had no effect on the number of tumors, but post-treatment increased the number and size of tumors. Kombairaju et al. reported that prolonged treatment with sulforaphane (0.5 mg, 5 days/week for 3 months did not improve tumorigenesis in the same LSL-K-rasG12D/+ murine model [125].
- A toxicity study of SFN supplementation carried out in mouse models showed that ingestion of high doses of sulforaphane produced: marked sedation (at 150–300 mg/kg), hypothermia (at 150–300 mg/kg), impaired motor coordination (at 200–300 mg/kg), decreased skeletal muscle strength (at 250–300 mg/kg) and deaths (at 200–300 mg/kg). In addition, blood analysis showed leukopenia in mice injected with sulforaphane at 200 mg/kg [118].
- Yagishita et al., 2019, provide an excellent review assessing the current state of knowledge on the relationships between formulation (e.g., plants, sprouts, drinks, supplements), bioavailability and efficacy, and the doses of glucoraphanin and/or sulforaphane that have been used in preclinical and clinical studies, paying particular attention to better integration of animal models and clinical studies, particularly with regard to dose selection and route of administration [126].

### 3.3. Optical Isomers of Sulforaphane

**Figure 17:**
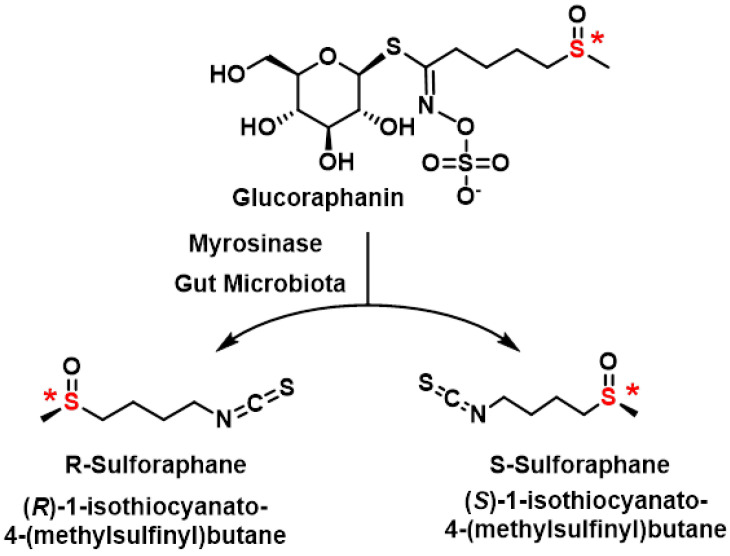
Chemical structures of glucoraphanin and enantiomers (R y S) of SFN. The stereogenic sulfur atom is indicated as S* and highlighted in red.

Glucoraphanin, the precursor of SFN found in broccoli, exists as a pure optical isomer with its sulfoxide group in the R configuration. This stereochemistry is preserved during the enzymatic hydrolysis by myrosinase, resulting in the formation of R-SFN. The sulfoxide group in SFN’s side chain contains a stereogenic sulfur atom that is configurationally stable, allowing for the separation of the two enantiomers (R and S) at room temperature [127], Figure 17.

SFN’s asymmetric sulfur atom gives rise to two optical isomers: R-SFN and S-SFN. However, in nature, specifically in broccoli sprouts, SFN exists exclusively as the R enantiomer. This natural occurrence of a single enantiomeric form is illustrated in Figure 16.

Research comparing these isomers has revealed significant differences in their biological activities. The naturally occurring R-SFN has demonstrated higher biological activity compared to its S counterpart. This finding has important implications for the potential therapeutic applications of SFN, as the R enantiomer may offer enhanced benefits [128].

The stereochemical aspects of SFN are crucial for understanding its biological effects and for developing potential pharmaceutical applications [129]. The higher activity of R-SFN suggests that maintaining this specific configuration could be important in the development of SFN-based therapies or supplements. Furthermore, this stereochemical preference in nature highlights the importance of chirality in biological systems and how subtle structural differences can lead to significant variations in biological activity [127].

Research by Vanduchova et al., 2019, highlights the distinct biological activities of SFN enantiomers, with the natural (R)-SFN showing significantly greater efficacy compared to the (S)-SFN enantiomer. Specifically, (R)-SFN enhances the activity of phase II detoxifying enzymes, such as quinone reductase and glutathione S-transferase, while (S)-SFN is either ineffective or much less active in this regard. This enantiomeric difference underscores the importance of stereochemistry in SFN’s biological activity [128].

Natural SFN predominantly exists as the (R)-enantiomer in broccoli, with its prevalence varying depending on the part of the plant and the variety of broccoli used for extraction. The content of (S)-SFN ranges from 1.5% to 41.8% of total SFN, depending on whether florets, leaves or stems are analyzed. For example, broccoli florets typically contain a higher proportion of (R)-SFN compared to stems and leaves [129].

The higher biological activity of (R)-SFN compared to its (S) counterpart has significant implications for its therapeutic potential. The ability of (R)-SFN to induce detoxifying enzymes makes it a promising candidate for cancer prevention and other health applications. These findings also emphasize the need for precise extraction and characterization methods to maximize the presence of the biologically active (R)-enantiomer in SFN-based products [130].

This variability in enantiomer content across different parts of broccoli highlights the importance of selecting specific plant parts and varieties for optimal SFN extraction. Additionally, advanced analytical techniques such as high-performance liquid chromatography (HPLC) are essential for accurately separating and quantifying SFN enantiomers, ensuring the efficacy and consistency of SFN-containing supplements and therapeutic formulations [131].

#### 3.3.1. Obtaining Sulforaphane Enantiomers

**Figure 18:**
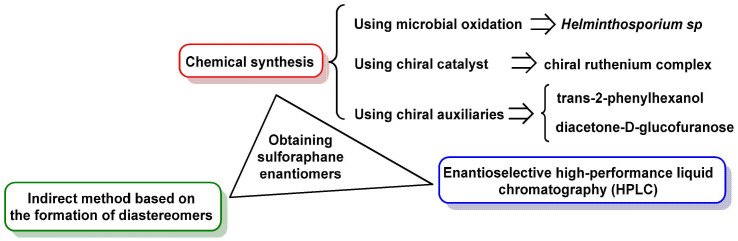
Methods for obtaining sulforaphane enantiomers.

The production of both enantiomers of SFN has been explored using a variety of methods, although only a few are described in the literature (Figure 18).

SFN naturally exists as a single enantiomer, specifically the (R)-SFN form, in broccoli and other Brassica plants. This enantiomer is responsible for the majority of SFN’s biological activity, including its antioxidant, anti-inflammatory, and anticancer properties. However, despite the prevalence of the natural (R)-SFN enantiomer, many studies investigating SFN’s biological activities have been conducted using the racemic form, which contains both (R)-SFN and (S)-SFN in equal proportions [132].

The use of racemic SFN in research introduces variability in results due to differences in biological activity between the two enantiomers. Studies have shown that (R)-SFN is significantly more effective at inducing phase II detoxification enzymes, such as quinone reductase and glutathione S-transferase, compared to (S)-SFN. The (S)-SFN enantiomer is either less active or completely inactive in these processes. As a result, the findings from studies using racemic SFN may not fully reflect the potency and efficacy of the naturally occurring (R)-SFN [98].

This discrepancy underscores the importance of conducting research specifically on the natural (R)-SFN enantiomer to better understand its unique biological effects. By focusing on the single enantiomer found in nature, researchers can more accurately evaluate its therapeutic potential and optimize its use in clinical applications. Additionally, isolating and studying (R)-SFN could lead to more targeted and effective formulations for dietary supplements and pharmaceutical products [133].

#### 3.3.2. Chemical Synthesis of Sulforaphane Enantiomers

Holland’s group achieved the production of (R)-SFN through microbial oxidation using *Helminthosporium* sp. to oxidize a prochiral sulfide precursor. This method resulted in an enantiomeric excess (ee) of 86% and a chemical yield of 54% [134].

**Figure 19:**
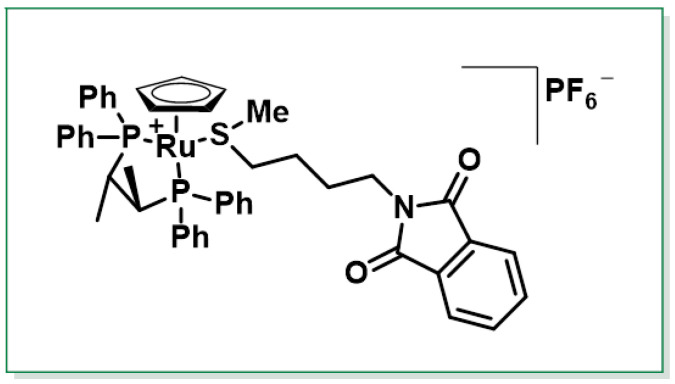
Diasteromeric intermediate used for the synthesis of enantioenriched R-SFN.

Another approach was described by Schenk et al., 1997, who developed an enantioselective synthesis of (R)-SFN with 80% ee. This method utilized a chiral ruthenium complex, specifically [CpRu((R,R)-CHIRAPHOS)]+, as a chiral catalyst. The process involved diastereoselective oxidation of a cationic ruthenium complex, providing an efficient route to the desired enantiomer [135], Figure 19.

The synthesis of SFN has been explored through various advanced methods, each aiming to achieve high yields, stereoselectivity and efficiency. One such method involves the oxygen transfer of dimethyldioxirane (DMD), which produces a sulfoxide complex with high throughput and excellent diastereoselectivity. In this approach, the cleavage of the phthaloyl group using aqueous hydrazine, followed by a reaction with thiophosgene, results in the formation of the SFN complex [CpRu(L–L)(MeS(O)C4H8NCS)]PF6. Finally, treatment of this complex with sodium iodide releases SFN without racemization, ensuring the retention of its enantiomeric purity [136,137].

These methods highlight the challenges and advancements in synthesizing enantiopure SFN. While microbial oxidation offers a biocatalytic approach, the use of chiral metal complexes enables precise control over stereochemistry. However, both methods have limitations in terms of yield and scalability, which may impact their practical application for large-scale production. 

Further research into novel synthetic strategies, such as the development of sulfinate ester intermediates or DAG (diacetone-d-glucofuranose) methodologies, could provide more efficient and versatile routes for producing SFN and its analogues. These approaches aim to improve yields, enantiopurity and scalability, making them promising candidates for future exploration in SFN synthesis [138].

**Figure 20:**
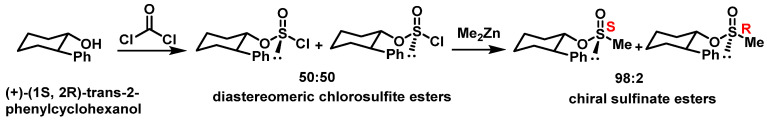
General procedures for the synthesis of chiral sulfinate esters via chlorosulfinates. In red, sulfur configuration in chiral sulfonate esters.

Another innovative method was developed by Whitesell et al., 1994, who used trans-2-phenylhexanol as a chiral auxiliary. The reaction of this chiral auxiliary with thionyl chloride produced an equimolar mixture of two diastereomeric chlorosulfite esters. Subsequent treatment of this mixture with dimethylzinc yielded both chlorosulfite esters with a favorable diastereomeric ratio and high chemical yields [139]. This process demonstrates the utility of chiral auxiliaries in achieving precise stereochemical control during SFN synthesis (Figure 20).

These methodologies highlight the advancements in synthesizing SFN with high stereoselectivity and chemical efficiency. The use of DMD for oxygen transfer ensures high diastereoselectivity, while Whitesell’s approach leverages chiral auxiliaries to optimize enantiomeric outcomes. Both methods contribute to the growing repertoire of techniques for producing SFN and its analogues, paving the way for their potential applications in pharmaceuticals and nutraceuticals.The method described was used to synthesize both enantiomers of SFN. The process for synthesizing (S)-SFN involves the following steps:

- Reaction of (+)-(1S,2R)-trans-2-phenylcyclohexyl(S)-methanesulfinate with a Grignard reagent derived from 4-chlorobutyl tert-butyldimethylsilyl ether.
- Removal of the tert-butyldimethylsiloxy protective group to yield (S)-4-hydroxybutyl methyl sulfoxide.
- Conversion of the alcohol to a mesylate.
- Reaction with sodium azide to form (S)-4-azidobutyl methylsulfoxide.
- Reaction of the azide with triphenylphosphine to form an iminophosphorane.
- Reaction of the iminophosphorane with carbon disulfide to produce enantiomerically pure (S)-SFN.

**Figure 21:**
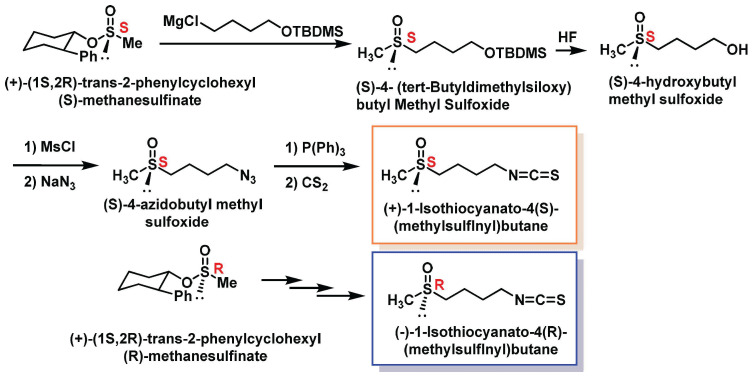
Synthesis of both enantiomers of SFN using (+)-(1S,2R)-trans-2-phenylcyclohexanol as a chiral auxiliary.

The same procedure was applied to the diastereoisomer (+)-(1S,2R)-trans-2-phenylcyclohexyl (R)-methanesulfinate to synthesize (R)-SFN. This enantio-divergent approach allows for the synthesis of both enantiomers of SFN using a single chiral auxiliary, demonstrating the versatility of the method. The process is illustrated in Figure 21.

**Figure 22:**
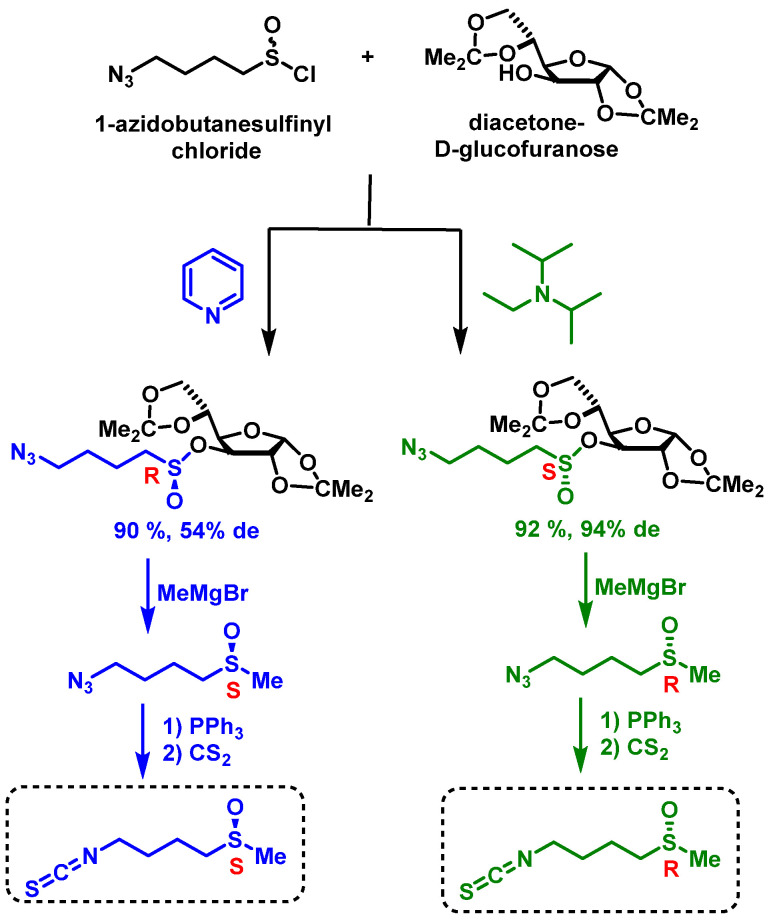
Enantio-divergent synthesis of both enantiomers of SFN.

The synthetic route provides a valuable method for producing both enantiomers of SFN, which is crucial for studying the biological activities of each isomer separately. Such enantioselective syntheses are important for developing potential therapeutic applications of SFN and its analogues. Khiar et al. developed an efficient method for synthesizing both enantiomers of SFN using diacetone-D-glucofuranose (DAG) as a chiral auxiliary. This approach, known as the “DAG methodology”, enables the stereoselective synthesis of both SFN enantiomers with high yields and enantiopurity. The process involves several key steps [140], Figure 22.

First, azidoalkanesulfinate chlorides react with DAG under different conditions to produce either (S)- or (R)-sulfinate esters. Using DIPEA as a base yields (S)-sulfinate esters with high yield and good diastereomeric excess, while pyridine as a base produces (R)-sulfinate esters with high yield but lower diastereomeric excess. The Grignard reaction is then employed to replace the chiral auxiliary in sulfinates, inverting the configuration at the sulfinyl sulfur. Finally, a Staudinger/aza-Wittig tandem reaction involving triphenylphosphine and carbon disulfide generates either R or S enantiopure SFN.

A notable feature of this method is its enantiodivergent nature. By simply changing the base used to catalyze the reaction, both epimers at sulfur become accessible through a dynamic kinetic transformation of the starting sulfinyl chloride. This flexibility makes the DAG methodology particularly versatile for producing SFN enantiomers [141].

The DAG methodology offers several advantages. It provides high yields and enantiopurity for both SFN enantiomers, allows for the synthesis of various sulfoxide stereoisomers, and can be adapted to produce SFN homologues and analogues. These features make it a valuable tool for researchers studying the biological activities of SFN and its derivatives or developing potential therapeutic applications [142,143].

This synthetic approach represents a significant advancement in SFN synthesis, offering researchers a powerful method for exploring its diverse biological functions and therapeutic potential.

#### 3.3.3. HPLC Separation of Sulforaphane Enantiomers in Broccoli

Enantioselective high-performance liquid chromatography (HPLC) has become the method of choice for analyzing natural and synthetic chiral compounds like SFN. This direct method employs chiral stationary phases to separate and quantify the R and S enantiomers of SFN [144].

Recent studies have demonstrated HPLC’s effectiveness in resolving racemic SFN. Mammone et al., 2024 [144], used the Chiralpak IH-3 chiral column containing amylose tris-[(S)-methylbenzylcarbamate] as the chiral selector. Using various mobile phases of pure alcoholic solvents and hydroalcoholic mixtures, they observed that (R)-SFN elutes before its S enantiomer. Panusa et al., 2020, investigated five immobilized-type amylose-based chiral stationary phases: Chiralpak IA-3, ID-3, IE-3, IF-3, and IG-3. With these columns, the elution order was reversed, with the S enantiomer eluting before the R enantiomer [145].

**Figure 23:**
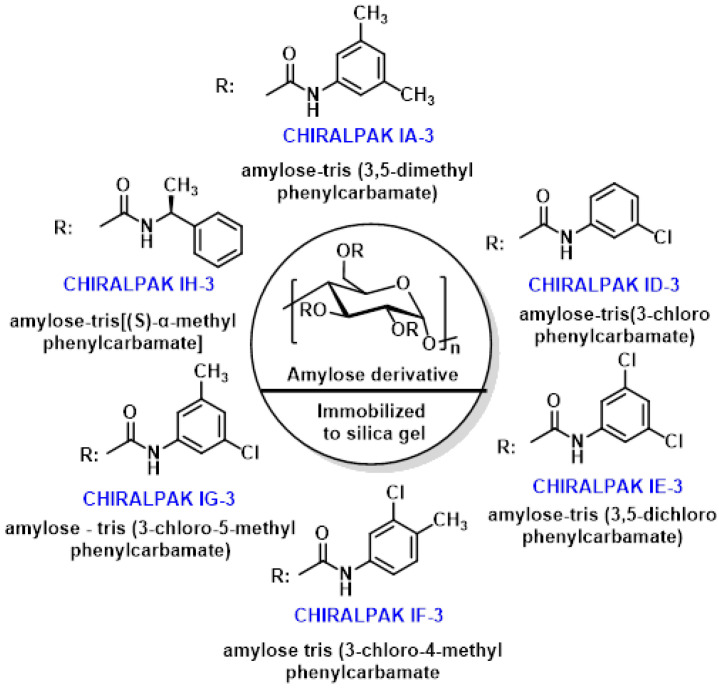
Amylose-based chiral stationary phases used in HPLC to discriminate SFN enantiomers.

The varying chiral resolution capabilities of these chiral stationary phases (CSPs) are attributed to the different substitution patterns in the aromatic ring of the amylose backbone (Figure 23).

These studies underscore the importance of selecting appropriate chiral columns and mobile phase conditions for accurate separation and quantification of SFN enantiomers. The ability to distinguish between R and S forms is crucial, as the R enantiomer is generally considered more biologically active.

The choice of chiral stationary phase significantly impacts the separation and elution order of SFN enantiomers. Researchers must carefully consider these factors when developing HPLC methods for SFN analysis to ensure accurate results and reliable quantification of the biologically active R enantiomer [146].

An alternative approach to separating SFN enantiomers involves an indirect method based on the formation of diastereomers. This technique relies on reacting the SFN enantiomers with a chiral derivatization reagent, creating diastereomers that can be separated using chromatography with achiral stationary phases [129].

**Figure 24:**
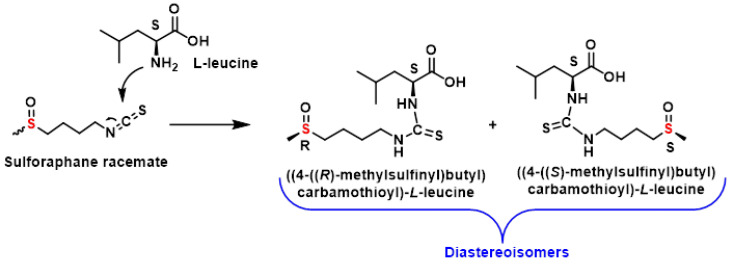
Formation of SFN diastereoisomers by (S)-Leucine reaction.

Okada et al., 2017, demonstrated this method by derivatizing racemic SFN with (S)-leucine. The resulting diastereomers were successfully resolved using reversed-phase high-performance liquid chromatography (HPLC) with UV detection. This approach provides a practical solution for determining the proportion of R and S enantiomers in SFN samples [129], Figure 24.

The indirect method offers several advantages over direct enantioselective techniques, such as chiral stationary phase-based HPLC. By forming diastereomers, this approach enables the use of standard achiral columns, which are often more accessible and cost-effective.

Additionally, it allows for precise quantification of trace amounts of one enantiomer in the presence of the other, making it particularly useful for studying enantiomeric purity. This method was used by Okada et al., 2017, who derived racemic SFN with (S)-leucine, and was resolved by reverse-phase HPLC with UV detection [129] (Figure 22).

This method highlights the versatility of chiral derivatization reagents in resolving racemic mixtures and underscores their importance in the analysis of SFN and other chiral compounds. As research continues to explore both direct and indirect methods, these techniques will remain essential tools for understanding the stereochemistry and biological activity of SFN enantiomers.

High-performance liquid chromatography (HPLC) represents the most popular and widely applicable technology in the field of chiral analysis of various chiral mixtures. Indirect separations are based on the formation of diastereoisomeric complexes between the enantiomers and suitable chiral derivatizing agents, and their subsequent separation by an achiral liquid chromatographic method. This method is not very practical, as derivatization represents an additional step that can lead to undesirable side reactions, the formation of decomposition products and racemization. In addition, the chiral derivatization reagent must be of high enantiomeric purity and the presence of derivatizable groups in the analyte is a prerequisite, and finally, once the diastereomers have been separated, the derivatization agent must be removed.

#### 3.3.4. Importance of Sulfur Chirality in the Biological Activity of SFN

Naturally occurring SFN exists exclusively as the R-enantiomer in terms of its sulfoxide sulfur configuration. However, most studies investigating its anti-inflammatory and antioxidant activities have been conducted using racemic mixtures. This discrepancy has led to evolving insights into the importance of SFN’s stereochemistry.

Initial findings suggested that sulfur chirality had no significant impact on SFN’s biological activity. However, recent discoveries have revealed important differences between the R and S enantiomers. In terms of enzyme induction, natural (R)-SFN has been shown to be a more potent inducer of carcinogen-detoxifying enzyme systems in rat liver and lung compared to the (S)-isomer [130].

Furthermore, (R)-SFN demonstrated superior ability in regulating two key enzyme systems involved in carcinogen metabolism. It increased microsomal epoxide hydrolase levels more effectively than the (S)-isomer and enhanced glucuronosyl transferase activity, while the (S)-enantiomer impaired it [147].

These findings highlight the importance of stereochemistry in SFN’s biological activities and suggest that the natural R-enantiomer may offer superior chemopreventive and detoxifying properties compared to its S counterpart or racemic mixtures. This understanding underscores the need for further research focusing on the specific biological activities of the R-enantiomer, which more closely resembles the naturally occurring form of SFN in broccoli and other cruciferous vegetables [147].

The evolving knowledge about SFN’s stereochemistry and its impact on biological activity has important implications for future research and potential therapeutic applications. It suggests that studies and interventions using pure (R)-SFN may yield more accurate and potentially more potent results than those using racemic mixtures. This insight could guide the development of more effective SFN-based therapies and dietary interventions [98].

## 4. Indole-3-Carbinol (I3C)

**Figure 25:**
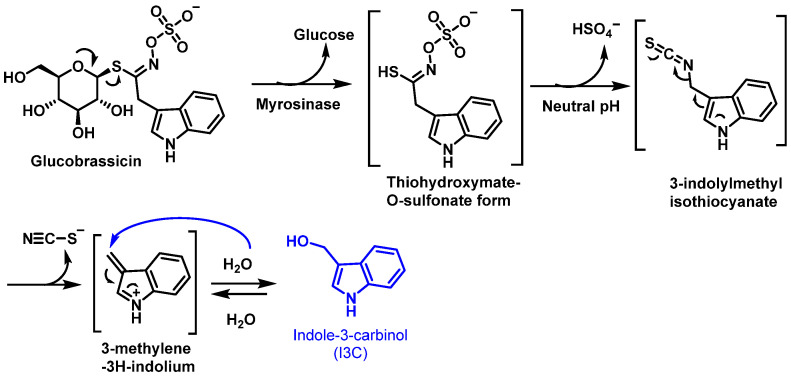
Biosynthesis of I3C starting from glucobrassicin.

Glucosinolate glucobrassicin, found in broccoli, is metabolized by the enzyme myrosinase into its corresponding isothiocyanate. This isothiocyanate undergoes thiocyanate removal, forming the intermediate 3-methylene-3H-indoleum. The intermediate then reacts with water to produce 3-methylene-3-carbinol, a phytochemical with a wide range of biological activities [59], Figure 25.

**Figure 26:**
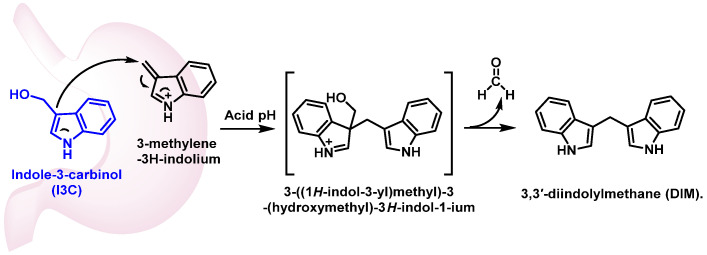
Formation of DIM in vivo by dimerization in acidic medium of indole-3-carbinol.

3-Methylene-3-carbinol exhibits cardioprotective, antioxidant, anti-inflammatory, antiangiogenic, and antimicrobial properties. Additionally, it promotes apoptosis in tumor cells, making it a compound of interest for potential therapeutic applications. These diverse effects highlight the importance of glucobrassicin-derived metabolites in health-promoting and disease-preventing mechanisms [148,149]. In vivo, 20 to 40% of this carbinol is transformed into 3,3′-diindolylmethane (DIM) by condensation in an acidic medium [150] (Figure 26).

Indole-3-carbinol (I3C) and its derivative diindolylmethane (DIM) exhibit anti-inflammatory properties through multiple mechanisms:

- Nrf2 activation: I3C positively stimulates the transcription factor Nrf2, which plays a crucial role in cellular defense against oxidative stress. Activation of the Nrf2-ARE signaling pathway by I3C and DIM leads to increased expression of cytoprotective genes, reducing inflammation and oxidative damage [151].
- NF-κB pathway inhibition: DIM’s main target is the NF-κB signaling pathway. By inhibiting this pathway, DIM decreases the production of proinflammatory cytokines such as TNF-α and IL-6, as well as prostaglandins. This results in a reduced inflammatory response [152].
- Modulation of inflammatory mediators: I3C treatment has been shown to decrease the expression of pro-inflammatory factors like IL-1β and IL-6 while increasing anti-inflammatory factors such as IL-4 and IL-10. This modulation of inflammatory mediators contributes to the overall anti-inflammatory effect [153].
- Regulation of microglia: I3C has been found to reduce the number of activated microglia and increase the number of M2-type microglia, which have anti-inflammatory properties. This regulation of microglia populations further contributes to the anti-inflammatory effects of I3C [154].
- Cancer chemoprevention: both I3C and DIM have been extensively studied for their chemopreventive properties against various types of cancer [155].

Their mechanisms of action include modulation of aryl hydrocarbon receptor (AHR) signaling, impact on estrogen receptor (ER)-dependent signaling in breast cancer cells, antagonism of the androgen receptor in prostate cancer, epigenetic regulation of gene expression and modulation of DNA methylation and histone modifications [156].

These diverse mechanisms highlight the potential of I3C and DIM as promising compounds for reducing inflammation and preventing cancer development.

## 5. Other Compounds in Broccoli

### 5.1. Polyphenols

Phenolic compounds are a major class of secondary metabolites in *Brassica* vegetables, including broccoli, where they play critical physiological roles. These compounds contribute to plant growth and reproduction and serve as a defense mechanism against pathogens, predators and ultraviolet radiation. Their high antioxidant capacity is attributed to their ability to act as hydrogen and electron donors, which enables them to neutralize reactive oxygen species (ROS). Additionally, phenolic compounds function as metal ion chelators, inhibiting the Fenton reaction and thereby reducing oxidative stress [157].

The flavonoid composition of broccoli inflorescences has been extensively studied using advanced techniques such as high-performance liquid chromatography coupled with mass spectrometry (LC/UV-DAD/ESI-MS). Broccoli contains derivatives of flavonols like quercetin, kaempferol, and, to a lesser extent, isorhamnetin. These flavonols are typically conjugated with sugars and organic acids, with glycosylation most frequently occurring at position 3 of the C-ring. However, substitutions can also occur at positions 5, 7, 3′, 4′, and 5′. The sugars involved in glycosylation are usually glucose or rhamnose, most commonly attached to position C3 and less frequently to C7 of ring A [158,159].

**Figure 27:**
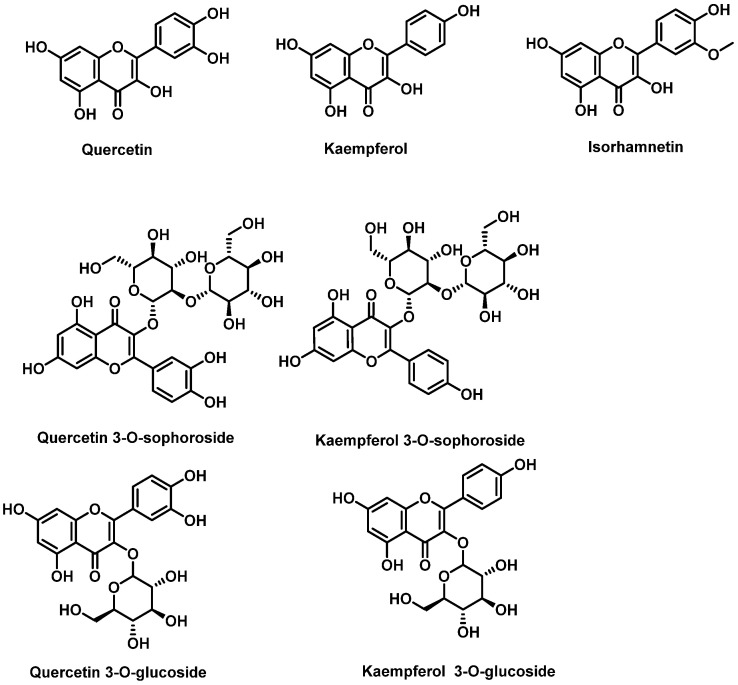
Most abundant flavanols in broccoli.

In *Brassica* crops, the most abundant glycosylated flavonols are quercetin 3-O-sophoroside and kaempferol 3-O-sophoroside. Other derivatives such as quercetin 3-O-glucoside and kaempferol 3-O-glucoside have also been identified. These compounds contribute significantly to the antioxidant properties of broccoli and other *Brassica* vegetables. Their ability to mitigate oxidative stress highlights their potential health benefits for humans while also playing crucial roles in the plant’s defense mechanisms [28,160] (Figure 27).

**Figure 28:**
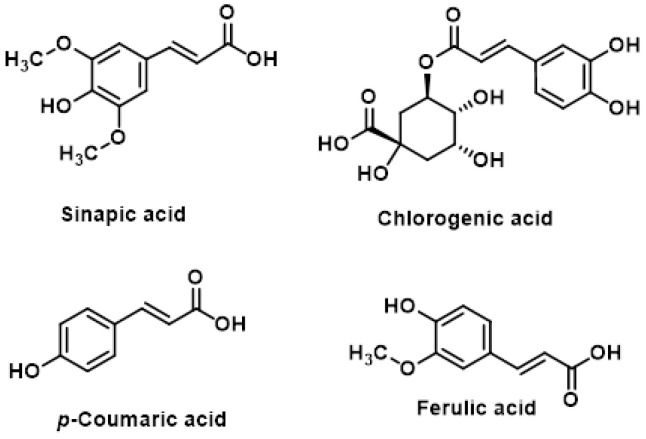
Some of the most common hydroxycinnamic acids in broccoli.

Hydroxycinnamic acids are a class of non-flavonoid phenolic compounds found in broccoli. These compounds and their derivatives include sinapic acid, chlorogenic acid, p-coumaric acid and ferulic acid [161] (Figure 28).

Recent studies have demonstrated that hydroxycinnamic acids and their derivatives exhibit anti-metastatic properties, particularly in inhibiting the spread of cancer cells to lungs and bones [162].

The anti-metastatic effects of hydroxycinnamic acids have been observed in various studies. p-hydroxycinnamic acid (HCA) has shown anticancer effects on MDA-MB-231 human breast cancer bone metastatic cells in vitro. HCA was found to suppress cancer cell proliferation, induce apoptosis in confluent cancer cells and prevent the negative effects of cancer cells on osteoblastogenesis and osteoclastogenesis. HCA’s anticancer effects are mediated through multiple signaling pathways, including NF-κB, extracellular signal-regulated kinase (ERK), protein kinase C, calcium signaling, phosphatidylinositol 3-kinase (PI3K) and nuclear transcription activity [163,164]. The apoptotic effects of HCA on cancer cells were found to be caspase-3 dependent, suggesting a mechanism involving the activation of this key apoptotic enzyme.

HCAs have demonstrated antioxidant properties, which may contribute to their anti-cancer effects. For example, quercetin 3-O-sophoroside, a flavonol glycoside found in broccoli, was shown to be a potent inhibitor of lipid peroxidation. Recent in silico studies have explored the potential of 4-hydroxycinnamic acid, found in broccoli, to interact with estrogen receptor beta protein, suggesting a possible role in hormone-related therapies [165].

These findings highlight the potential of hydroxycinnamic acids and their derivatives as promising candidates for the prevention and treatment of cancer metastasis, particularly in breast cancer bone metastasis [166].

### 5.2. Carotenoids

Carotenoids are tetraterpene fat-soluble pigments composed of 40 carbon atoms arranged in conjugated polyenic chains. Humans cannot synthesize carotenoids, so they must be obtained through diet or supplements. The major carotenoids found in the genus *Brassica*, including broccoli, are β-carotene, lutein, and neoxanthin. *Broccoli* sprouts can contain up to 1.16 mg of carotenoids per 100 g of fresh weight [167]. These compounds act as potent antioxidants, helping to protect cells from damage caused by free radicals, particularly in tissues such as the eyes and skin [168]. Carotenoids exhibit several important functions:

- In eye health, lutein and zeaxanthin form the macular pigment in the retina, protecting photoreceptor cells from oxidative stress caused by sunlight exposure. This may help reduce the risk of age-related macular degeneration [169].
- In skin protection, dietary carotenoids accumulate in the skin and offer measurable photo-protective benefits against UV-induced damage. Carotenoids can quench singlet oxygen and scavenge toxic free radicals, preventing or reducing oxidative stress [170].
- Some carotenoids, like β-carotene, serve as precursors to vitamin A, which is essential for various biological processes, including vision [171].

**Figure 29:**
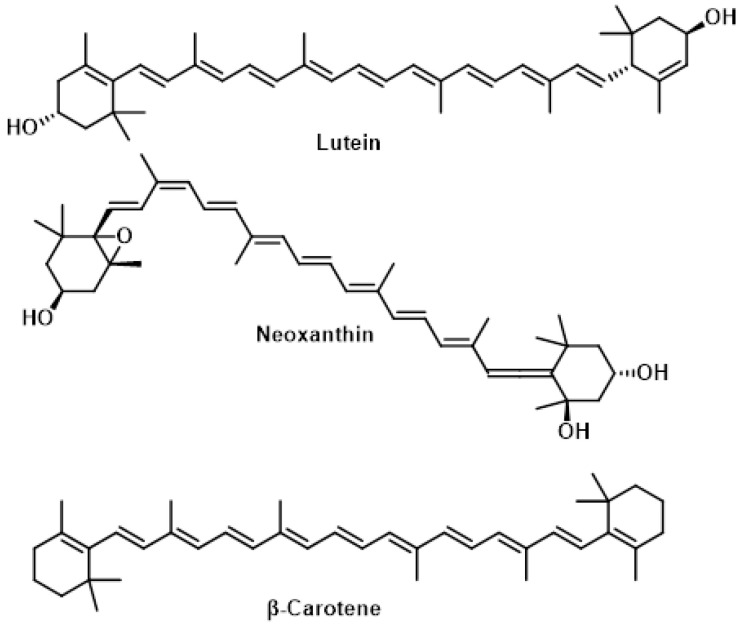
Chemical structure of carotenoids in broccoli.

Recent research has shown that the antioxidant properties of carotenoids can vary depending on oxygen concentration, potentially switching from antioxidant to pro-oxidant behavior. This highlights the complexity of carotenoid function in the body and the need for further research to fully understand their health benefits (Figure 29) [172].

Carotenoids, including lutein, neoxanthin and β-carotene, are important compounds found in broccoli that exhibit lipophilic antioxidant and immunomodulatory activities. Their biological effects stem from the conjugated double bonds in their long polyene chain, which enable them to inhibit reactive oxygen species and reduce oxidative damage [173]. The distribution of carotenoids varies across different parts of the broccoli plant [174]:

- Florets and leaves, that contain high levels of lutein, neoxanthin, and β-carotene.
- Stems, that contain lutein and neoxanthin, but lack β-carotene.

Lutein is particularly abundant in broccoli, and its concentration can increase when various cooking methods are applied, compared to fresh broccoli. This suggests that certain cooking techniques may enhance the bioavailability of some carotenoids. The carotenoid content in broccoli contributes to its health benefits due to antioxidant activity, neutralizing harmful free radicals and reduce oxidative stress. Lutein is important for eye health and may help prevent age-related macular degeneration [175].

Interestingly, broccoli leaves, which are often discarded, contain the highest concentrations of carotenoids, especially β-carotene. This finding suggests potential applications for broccoli by-products in enhancing the nutritional value of food products or as sources of bioactive compounds [176].

### 5.3. Tocopherols

In broccoli, tocopherols, which are forms of vitamin E, play an important role as lipid-soluble antioxidants that protect cell membranes from oxidative damage. Among the tocopherols present in broccoli, α-tocopherol is the most abundant, accounting for 78.5% of the total tocopherol content. This makes α-tocopherol the primary contributor to broccoli’s vitamin E activity, as it is the most biologically active form of tocopherol in humans and is essential for maintaining cellular health and preventing oxidative stress [177].

The remaining tocopherols are present in smaller proportions. γ-tocopherol constitutes 19.3% of the total tocopherols and, while less biologically active than α-tocopherol, it has unique antioxidant properties, particularly in scavenging reactive nitrogen species. δ-tocopherol, which accounts for 2.2% of the total tocopherols in broccoli, also contributes to antioxidant defense but is present in much lower concentrations [178].

The predominance of α-tocopherol in broccoli highlights its potential role as a dietary source of vitamin E, which is crucial for protecting lipids, proteins, and DNA from oxidative damage. The presence of γ- and δ-tocopherol adds to the diversity of antioxidant compounds in broccoli, further enhancing its health-promoting properties. The balance of these tocopherols may vary depending on factors such as broccoli variety, growing conditions, and post-harvest handling, but their combined presence underscores broccoli’s value as a nutrient-dense vegetable with significant antioxidant capacity [178].

### 5.4. Vitamin C

Broccoli stands out as an excellent source of vitamin C, boasting approximately 100 mg per 100 g fresh weight. This impressive content significantly surpasses that of other cruciferous vegetables like cabbage or cauliflower, which contain between 35 and 68 mg per 100 g fresh weight [15].

The vitamin C in broccoli serves as a potent antioxidant, effectively scavenging free radicals and shielding cells from oxidative damage. It also plays a crucial role in regenerating other antioxidants in the body, such as vitamin E [179,180,181,182,183]. Notably, the antioxidant activity of vitamin C is dose-dependent, with higher doses exhibiting more pronounced antioxidant effects [184].

Several factors influence the vitamin C content in broccoli. Genetic variability can lead to up to 71% variation in vitamin C levels among different broccoli varieties. Environmental factors, such as sunlight exposure also impact vitamin C content. Additionally, harvesting and storage processes can affect the final vitamin C levels in the vegetable [38].

Cooking methods significantly influence the retention of vitamin C in broccoli. Steaming emerges as the best method for preserving vitamin C, while boiling and stir-frying can result in substantial losses. Despite these potential losses during cooking, broccoli remains an excellent source of vitamin C, providing up to 101% of the daily recommended value in a 100 g serving [185].

In conclusion, broccoli’s high vitamin C content, coupled with its resilience to some degree of processing, makes it a valuable addition to a healthy diet, offering substantial antioxidant benefits even after cooking.

### 5.5. Phytosterols

Phytosterols, natural compounds found in plants, exhibit anti-inflammatory properties and have been associated with various health benefits, particularly in cardiovascular health. These compounds have been linked to reduced risks of cardiovascular disease and heart attacks, as well as helping maintain healthy blood cholesterol levels [186].

A study conducted by Shi et al., 2024, investigated the composition of bioactive compounds in broccoli heads, considering different organ sizes and growing seasons. Their research identified six phytosterols present in broccoli heads: beta-sitosterol, stigmasterol, campesterol, beta-sitosterol acetate, lanosterol, and cholesterol [187].

**Figure 30:**
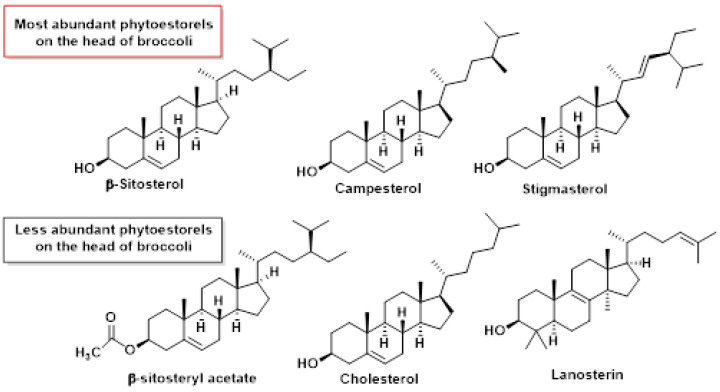
Chemical structure of phytosterols in the head of broccoli.

Among these phytosterols, beta-sitosterol was found to be the most abundant, with content ranging from 5.67 to 20.78 mg per 100 g of broccoli. Campesterol was the second most prevalent, with concentrations between 0.7 and 6.37 mg per 100 g, followed by stigmasterol, ranging from 0.48 to 2.38 mg per 100 g. Interestingly, beta-sitosterol acetate, lanosterol, and cholesterol were present in such small quantities that they were nearly undetectable in the broccoli heads [187] (Figure 30).

The presence of these phytosterols, particularly beta-sitosterol, contributes to broccoli’s potential health benefits. Phytosterols have been shown to help lower LDL cholesterol levels, with some studies suggesting that consuming 2 g of phytosterols daily may reduce LDL cholesterol by up to 10% [188].

Phytosterol content in broccoli can vary depending on factors such as growing conditions, plant part, and preparation methods. For instance, cooked broccoli may have different phytosterol concentrations compared to raw broccoli [187].

These findings highlight broccoli’s role as a valuable source of phytosterols in the diet, potentially contributing to its overall health-promoting properties. The presence of these beneficial compounds further underscores the importance of including broccoli in a balanced diet for maintaining cardiovascular health and managing cholesterol levels [189].

## 6. Antimicrobial Peptides

Antimicrobial peptides (AMPs) derived from broccoli (*Brassica oleracea* var. *italica*) have emerged as promising bioactive molecules, offering natural solutions to combat microbial infections and address various health challenges [190,191]. These small, proteinaceous molecules exhibit broad-spectrum activity against bacteria, fungi, and yeasts, making them valuable candidates for therapeutic applications [192].

### 6.1. Distribution and Sources of Broccoli AMPs

Broccoli contains a diverse array of AMPs distributed across its various parts, including seeds, stems, leaves, and fermented extracts. These peptides are integral to the plant’s defense system, protecting it from microbial attack while also offering significant potential for human health applications [193].

Recent studies have identified a range of peptides from different parts of broccoli, each with unique properties and mechanisms of action (Table 2).

The bioactivity of these peptides extends beyond antimicrobial effects to include anti-inflammatory, antioxidant, and enzyme inhibitory properties [15]. Pacheco-Cano et al. (2020) identified Class I defensins (BraDef) from broccoli seeds, which demonstrated potent antimicrobial activity. These small, cysteine-rich peptides play a crucial role in plant defense mechanisms and show promise for applications in human health [192].

Focusing on broccoli stems, Nicolas-Espinosa et al. (2022) explored bioactive peptides that enhance wound healing and keratinocyte proliferation. Their comprehensive study identified 1256 peptide sequences in protein extracts from broccoli stems, highlighting the rich diversity of bioactive compounds in this often-overlooked part of the plant [194]. Similarly, Chen et al. (2020) investigated peptides from broccoli stems and leaves, concentrating on their antioxidant and hypolipidemic properties. Their findings suggest potential applications in managing oxidative stress and lipid metabolism, expanding the therapeutic potential of broccoli-derived peptides beyond antimicrobial applications [193].

The antimicrobial activities of peptides isolated from cruciferous vegetables, including broccoli, were explored by Favela-González et al., emphasizing their potential as natural alternatives to synthetic antimicrobials [198]. This research underscores the growing interest in plant-derived AMPs as sustainable solutions to combat microbial resistance and promote human health. Furthermore, Li et al. (2023) identified novel anti-inflammatory peptides from broccoli fermented by Lactobacillus strains, employing virtual screening and molecular docking techniques [196]. This innovative approach to identifying bioactive peptides opens new avenues for discovering and developing broccoli-derived compounds with specific therapeutic properties.

### 6.2. Mechanisms of Action

The antimicrobial peptides (AMPs) derived from broccoli exhibit diverse mechanisms of action, targeting bacterial membranes, enzymes, oxidative stress pathways, and inflammatory mediators. These multifaceted approaches contribute to their effectiveness against a wide range of pathogens and their potential therapeutic applications.

Membrane disruption is a primary mechanism employed by several broccoli-derived AMPs. Pacheco-Cano et al. (2020) identified that peptides like ARFEELNMDLFR, isolated from broccoli stems, create pores in bacterial cell membranes, leading to ion leakage and cell lysis [192]. This mechanism is particularly effective against gram-negative bacteria such as *Escherichia coli*. Additionally, SFN disrupts biofilm formation in pathogens like *Helicobacter pylori*, preventing microbial adhesion to host tissues and enhancing the overall antimicrobial effect [199].

Enzyme inhibition represents another crucial mechanism through which broccoli-derived peptides exert their effects. Anti-inflammatory peptides like SIWYGPDRP bind to inducible nitric oxide synthase (iNOS), effectively reducing NO production in macrophages and contributing to their anti-inflammatory properties.

The anti-inflammatory effects of broccoli-derived AMPs extend beyond oxidative stress modulation. Li et al. (2023) isolated the peptide RFR from fermented broccoli, which demonstrated a remarkable 75% inhibition of TNF-α release [196]. This potent anti-inflammatory action suggests potential applications in managing chronic inflammatory diseases such as rheumatoid arthritis or inflammatory bowel disease.

Recent studies have also revealed that some AMPs can target intracellular components of pathogens. While specific examples from broccoli are yet to be fully elucidated, this mechanism involves AMPs binding to nucleic acids and proteins within microbial cells, impeding replication and other essential cellular processes [200]. This intracellular targeting adds another layer to the antimicrobial arsenal of broccoli-derived peptides.

### 6.3. Potential Applications

**Figure 31:**
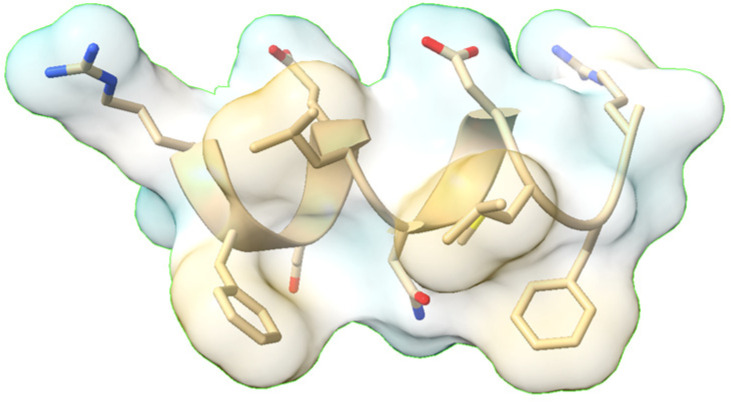
Broccoli antimicrobial peptide ARFEELNMDLFR. Structure was modeled using Alphafold2. Image was generated using the software ChimeraX 1.9. The peptide is displayed in stick representation, with a transparent hydrophobic surface overlay colored using a gradient from blue (hydrophilic) to yellow (hydrophobic).

The antimicrobial peptides (AMPs) derived from broccoli hold transformative potential across multiple sectors, offering eco-friendly solutions to pressing global challenges. In the food industry, broccoli extracts are emerging as natural preservatives to combat foodborne pathogens. Studies demonstrate that peptides like ARFEELNMDLFR (Figure 31) inhibit *Salmonella* and *Listeria monocytogenes*, reducing spoilage and extending shelf life [194,195]. 

Pacheco-Cano et al. (2020) showed that broccoli seed defensins (BraDef) effectively suppress *Bacillus cereus* [192], a common contaminant in dairy and meat products. Innovators are now integrating antimicrobial peptides into edible films and packaging materials, creating “active packaging” that passively releases antimicrobial agents to protect perishable goods. This approach not only enhances food safety but also aligns with consumer demand for clean-label, synthetic-free products [201,202].

In agriculture, broccoli AMPs offer a sustainable alternative to chemical pesticides. Nicolas-Espinosa et al., 2022, highlighted that stem-derived peptides suppress phytopathogenic fungi such as *Aspergillus niger* and *Colletotrichum gloeosporioides*, which devastate crops like tomatoes and citrus [194]. Additionally, these peptides are biodegradable, minimizing environmental runoff and soil toxicity compared to conventional fungicides [203].

**Figure 32:**
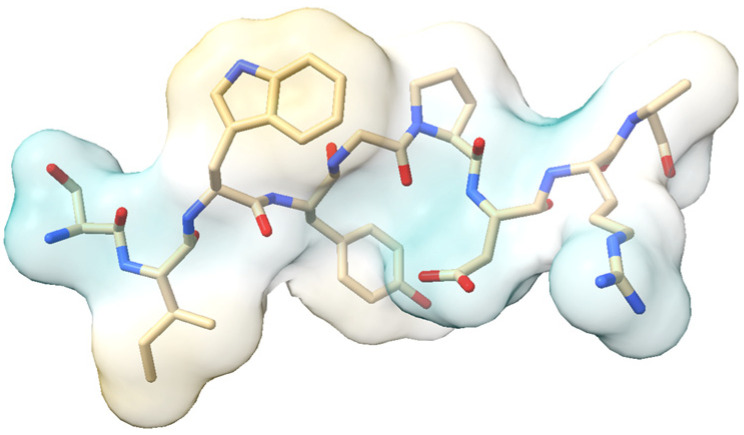
Broccoli anti-inflammatory peptide SIWYGPDRP. Structure was modeled using Alphafold2. Image was generated using the software ChimeraX 1.9. The peptide is displayed in stick representation, with a transparent hydrophobic surface overlay colored using a gradient from blue (hydrophilic) to yellow (hydrophobic).

There has been a growing interest in the health benefits of plants, driven by their safety and versatility in developing pharmaceuticals and cosmetics [204]. The healthcare sector benefits from broccoli AMPs’ dual antimicrobial and regenerative properties. For example, peptides from fermented broccoli stems accelerate wound closure by stimulating keratinocyte migration, as shown in murine models where treated wounds healed 40% faster than controls [194]. SFN has shown promise against antibiotic-resistant pathogens like MRSA and *Helicobacter pylori*, the latter being a major cause of gastric ulcers [205]. Li et al. (2023) identified anti-inflammatory peptides such as SIWYGPDRP (Figure 32), which inhibits nitric oxide (NO) production by 52%, offering potential therapies for chronic inflammation and autoimmune disorders [196].

Beyond these fields, cosmeceuticals are leveraging broccoli peptides for skincare. Peptide mixtures from stems enhance collagen synthesis and protect against UV-induced oxidative stress, making them ideal for anti-aging formulations. Chen et al. (2020) further reported hypolipidemic effects in broccoli leaf peptides, suggesting applications in functional foods targeting metabolic syndrome [193]. Broccoli extract is also a potential anti-photoaging agent [206]. As research in this field progresses, a deeper understanding of these mechanisms will likely lead to novel therapeutic strategies and applications in both human health and agriculture.

## 7. Use of Broccoli By-Products as a Source of Bioactive Products

An important trend in the future will be to consider the revaluation of broccoli by-products. Only a small proportion of broccoli is used in food, the remaining stems, leaves, roots and inflorescences are discarded, which leads to an increase in broccoli by-products that are mostly used for composting or incorporated into the soil [207]. These by-products can also be rich in bioactive compounds and present beneficial properties [208,209,210]. Current research on the functionality of broccoli by-products, such as leaves and stems, has provided valuable information on the use of these waste materials as new ingredients for the food, cosmetic and/or pharmaceutical industry.

As ingredients in functional foods [176], to improve the nutritional properties of foods [211] or as natural preservatives to prolong their shelf life, it is of great importance to consider the best use of plant foods at a time when the hectares of arable land are decreasing and the world’s population is increasing [212].

In the pharmaceutical industry, these by-products offer considerable health benefits due to their richness in bioactive compounds, similar to the edible parts. Studies have shown that by-products such as stems and leaves contain high levels of phenolic compounds, glucosinolates, vitamins and fiber [213,214,215].

In the cosmetics industry, consumers are more inclined to choose products of natural origin, so the use of by-products from broccoli by-products would be a great source of bioactive compounds for these products. This is why more and more people are looking to incorporate these elements into the development of new cosmetic products and the cosmetic industry may represent a cost-effective solution on how to recycle disposable by-products [216].

## 8. Conclusions

Broccoli, a widely consumed vegetable from the Brassica genus, has been extensively studied for its health benefits, with new advantages continually being discovered. The nutritional and health-promoting properties of broccoli are largely attributed to its rich content of glucosinolates (GSLs), secondary metabolites abundant in nitrogen and sulfur. GSLs have demonstrated protective properties against various diseases, particularly cancer. These compounds contribute to improving human health through several mechanisms, such as anti-inflammatory effects, antioxidant activity (broccoli is rich in antioxidants, including vitamin C, carotenoids, and anthocyanins, which help combat oxidative stress), cardiovascular health and metabolic regulation of glucose and lipid metabolism, potentially reducing the risk of type 2 diabetes and metabolic syndrome. SFN, a key isothiocyanate derived from broccoli, has garnered significant attention for its health-promoting properties: anticancer activity against various types of cancer, including prostate, pancreatic, leukemia, and colon cancer; enhancing the body’s antioxidant defenses by activating the Nrf2 pathway and NF-κB inhibition. Despite the promising health benefits of SFN, several challenges and areas for future research remain: chemical instability, the clinical use of SFN has been hampered by its chemical instability, necessitating the development of more stable formulations. SFN has optical isomers due to the chirality of sulfur, clinical and toxicological studies are necessary to fully evaluate the effects of SFN enantiomers on different organs of the human body. Appropriate enantio-selective synthesis methods and environmentally friendly solvent HPLC enantio-separation methods are needed.

The antimicrobial peptides (AMPs) derived from broccoli exhibit diverse mechanisms of action, targeting bacterial membranes, enzymes, oxidative stress pathways, and inflammatory mediators. These multifaceted approaches contribute to their effectiveness against a wide range of pathogens and their potential therapeutic applications.

In conclusion, while broccoli and its bioactive compounds, particularly GSLs and SFN, show great promise in promoting human health and preventing chronic diseases, further research is needed to overcome the challenges associated with their stability, synthesis, and clinical application. Additionally, more comprehensive human trials are required to establish optimal consumption patterns and to fully elucidate the mechanisms by which these compounds exert their beneficial effects.

## Figures and Tables

**Table 1:** Major anticancer mechanisms of SFN and concentrations needed for triggering them.

Mechanism	Concentration	Concentration
	In Vitro	In Vivo
Modulation of phase I and phase II enzymes	0.5–25 μM	6 μmol/day to 1 mmol/kg
DNA protection from chemical insult	0.06–20 μM	10–450 μmol/kg
Induction of apoptosis	0.1–300 μM	2.4 μmol to 15 nmol/day
Cell-cycle modulation	<1 μM–2 mM	10–100 μmol/day
Inhibition of angiogenesis	0.1–50 μM	
Inhibition of metastasis formation	6–28 μM	2.8 μmol/kg

**Table 2:** Antimicrobial and antioxidant peptides and proteins from broccoli.

Peptide Name/Sequence	Activity	Target Pathogens/Effects	Reference
ARFEELNMDLFR	Antimicrobial activity	*Porphyromonas gingivalis*, *Candida albicans*	[194,195]
SIWYGPDRP	Anti-inflammatory (52% NO inhibition)	Reduces inflammation in macrophages	[196]
RFR	TNF-α inhibition (75%)	Chronic inflammatory diseases	[196]
KASFAFAGL	IL-6 inhibition (30%)	Autoimmune disorders	[196]
KSVLLKF	Antioxidant, hypolipidemic	Oxidative stress, lipid metabolism	[193]
BoNap	Antifungal inhibition	*Fusarium culmorum*, *Penicillium expansum*	[197]
BraDef (Class I)	Broad-spectrum antimicrobial	*B. cereus*, *C. albicans*	[192]

## Data Availability

No new data were created or analyzed in this study. Data sharing is not applicable to this article.
